# Pulsed Field Ablation Versus Thermal Ablation for First‐Time Pulmonary Vein Isolation in Atrial Fibrillation: A Systematic Review and Meta‐Analysis

**DOI:** 10.1002/joa3.70438

**Published:** 2026-08-06

**Authors:** Yousef Radwan Alnomani, Abdelaziz A. Awad, Ahmed Abdelaziz, Mazen Negmeldin Aly Yassin, Omnia Samy El‐Sayed, Menna Marwan, Alaa Radwan Mady, Mahmoud Ali, Khaled Akil, Belal Hamed, Al‐Hassan Soliman Wadan, Mohamed Abdelsalam

**Affiliations:** ^1^ Faculty of Medicine Benha University Benha Egypt; ^2^ Medical Research Group of Egypt (MRGE) Cairo Egypt; ^3^ Faculty of Medicine Al‐Azhar University Cairo Egypt; ^4^ Division of Cardiology Montefiore Health System/Albert Einstein College of Medicine Bronx New York USA; ^5^ Faculty of Medicine Cairo University Cairo Egypt; ^6^ Faculty of Medicine Zagazig University Zagazig Egypt; ^7^ Faculty of Medicine Port Said University Port Said Egypt; ^8^ Faculty of Pharmacy Menoufia University Shebin El‐Kom Egypt; ^9^ Faculty of Medicine Al‐Azhar University Damietta Egypt; ^10^ Department of Internal Medicine (Nephrology) Aleppo Hospital for Internal Diseases (formerly Zahi Azrak Hospital) Aleppo Syria; ^11^ Oral Biology Department, Faculty of Dentistry Galala University Suez Egypt; ^12^ Faculty of Medicine Misr University for Science and Technology Giza Egypt

**Keywords:** atrial fibrillation, catheter ablation, cryoballoon ablation, pulmonary vein isolation, pulsed‐field ablation, radiofrequency ablation

## Abstract

**Background:**

With over 60 million cases worldwide, atrial fibrillation (AF) is one of the most common cardiac arrhythmias in adults. Pulsed‐field ablation (PFA) has emerged as a significant alternative to thermal ablation for pulmonary vein isolation (PVI). This systematic review and meta‐analysis investigated the efficacy and safety of PFA versus thermal ablation in patients undergoing first‐time PVI.

**Methods:**

We systematically searched PubMed, Scopus, Web of Science, and Cochrane Library from inception to 2025. Hazard ratios (HRs) with 95% confidence intervals (CIs) were derived from reconstructed Kaplan–Meier survival curves to compare atrial tachyarrhythmia recurrence between PFA and thermal ablation. Random‐effects models were used to compare AF recurrence, durable PVI, redo procedures, phrenic nerve palsy (PNP), and other safety outcomes between the two groups.

**Results:**

A total of 27 studies comprising 8546 patients were included. Kaplan–Meier reconstruction from ten studies demonstrated higher 12‐month arrhythmia‐free survival with PFA compared with thermal ablation (78.4% [95% CI, 76.1–80.8] vs. 73.4% [95% CI, 71.5–75.4]). Thermal ablation was associated with a significantly higher risk of atrial tachyarrhythmia recurrence (HR 1.28, 95% CI 1.10–1.48; *p* = 0.001). Subgroup analyses demonstrated that PFA significantly reduced the recurrence risk in patients with paroxysmal AF (HR 1.63, 95% CI 1.31–2.03; *p* < 0.001), whereas no significant difference was observed in those with persistent AF (HR 1.13, 95% CI 0.92–1.41; *p* = 0.25).

**Conclusions:**

PFA was associated with lower rates of atrial tachyarrhythmia and AF recurrence rates than thermal ablation in patients undergoing first‐time PVI; however, these findings should be interpreted cautiously, given the predominance of observational studies.

## Graphical abstract

In patients undergoing first‐time pulmonary vein isolation for atrial fibrillation, pulsed field ablation was associated with lower arrhythmia recurrence and reduced transient phrenic nerve palsy compared with thermal ablation. Durable pulmonary vein isolation and most safety outcomes were similar between treatment strategies.

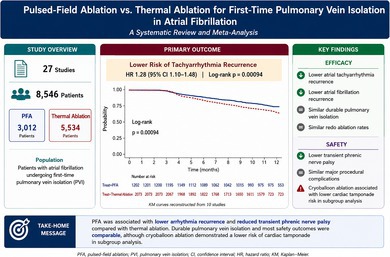

**Abbreviations**
CBA: cryo‐balloon ablation
CENTRAL: Cochrane Library Central Register of Controlled Trials
CI: confidence interval
HR: hazard ratio
*I* ^2^
: inconsistency test
MD: mean difference
NOS: the New Castle Ottawa Scale
OR: odds ratio
PFA: pulsed field ablation
PRISMA: Preferred Reporting Items for Systematic Reviews and Meta‐Analyses
PVI: pulmonary vein isolation
RFA: radiofrequency ablation
ROB‐2: Risk of Bias 2 tool
ROBINS‐1: risk of bias in nonrandomized studies of interventions
RR: risk ratio

## 1 Introduction

Atrial fibrillation (AF) is the most common cardiac arrhythmia in adults, with an estimated prevalence of about 60 million cases worldwide [1]. AF is associated with reduced quality of life and increased risk of major cardiovascular morbidities, such as stroke, congestive heart failure, and sudden cardiac death [2]. These adverse outcomes represent a substantial burden to healthcare systems and public health and underline the requirement for effective management strategies. Catheter ablation is a safe and effective method of managing AF. It is associated with reduced mortality both in patients with AF and in patients with heart failure with reduced ejection fraction when compared with medical therapy. It also reduces cardiovascular hospitalizations and atrial arrhythmia recurrence in patients with AF, with the greatest benefits seen in men and those under 65 years of age [3, 4]. Pulmonary vein isolation (PVI) is a well‐established technique for the management of AF by rhythm control and is a form of catheter ablation [5]. PVI is mainly performed by thermal ablation techniques, including radiofrequency ablation (RFA) and cryoballoon ablation (CBA). Thermal ablation, however, lacks tissue selectivity and may cause collateral tissue damage and severe complications, such as pulmonary vein stenosis and atrio‐esophageal fistula [6, 7].

Pulsed‐field ablation (PFA) is a newer, non‐thermal, tissue‐specific alternative to thermal PVI techniques [8]. PFA applies ultrarapid, high amplitude electrical fields to targeted cardiac tissues and creates pores in the cardiomyocyte cell membrane. This induces cardiomyocyte apoptosis without collateral tissue damage, a major advantage compared to thermal‐based PVI methods [9]. The PFA technique has promising results, but clinical studies have produced conflicting results. For instance, Maurhofer et al. [10] demonstrated that PFA for PVI versus thermal ablation was related to one‐year freedom from any atrial arrhythmia. Conversely, Della Rocca et al. [11] did not find any significant difference in 1‐year freedom from any atrial tachyarrhythmia between PFA and thermal ablation. The potential advantages of the PFA technique and the inconsistent findings of current clinical studies underline the need for a comprehensive comparison of PFA and thermal ablation. The aim of this systematic review and meta‐analysis was to conduct a comprehensive and robust comparison of the PFA and thermal ablation techniques in patients with AF undergoing first‐time PVI addressing these inconsistencies.

## 2 Methods

We conducted this systematic review and meta‐analysis in accordance with the guidelines outlined in the Preferred Reporting Items for Systematic Reviews and Meta‐Analysis (PRISMA) [12]. The method was conducted in accordance with the Cochrane Handbook for Systematic Reviews and Meta‐Analysis of Interventions (version 5.1.0). The study protocol was registered on PROSPERO (CRD42024527990).

### 2.1 Eligibility Criteria

We included all clinical studies comparing PFA with thermal ablation (CBA or RFA) in patients with AF undergoing first‐time PVI, assessing the following outcomes: atrial tachyarrhythmia recurrence, AF recurrence, durable PVI, redo procedure, transient and persistent peripheral nerve palsy (PNP), cardiac tamponade, esophageal injury, groin complications, vascular access complications, thromboembolic events, procedure duration, skin‐to‐skin duration, left atrial dwell time, and fluoroscopy time. We excluded studies that included patients with previous ablation, as well as research letters and non‐English publications.

### 2.2 Literature Search

We conducted a systematic search on the PubMed, Scopus, Web of Science, and Cochrane Library databases for all published studies until February 2025. Our search strategy included the following main terms: (“pulsed field ablation” OR “PFA”) AND (“thermal ablation” OR “radiofrequency ablation” OR “RFA” OR “cryo‐balloon ablation” OR “CBA”) AND (“atrial fibrillation” OR “AF”). The search strategies used for each database are detailed in Supplementary Table 1.

### 2.3 Screening of the Literature Search Results

All studies that appeared in the database search underwent title and abstract screening to determine their eligibility for inclusion. Potentially relevant studies were retrieved for full‐text screening, and those that met our inclusion criteria were included. We conducted a citation analysis by searching the references of all the included studies for any additional eligible studies.

### 2.4 Data Extraction

A standardized data extraction sheet was used to record the data extracted from the included studies. Data were extracted into the following categories: (1) study characteristics (year of publication, study design, PFA and thermal ablation techniques and procedural details, follow‐up duration, location, and database source), (2) characteristics of the study participants, (3) risk of bias assessment, and (4) outcomes of interest (atrial tachyarrhythmia recurrence, AF recurrence, durable PVI, redo procedure, transient and persistent PNP, cardiac tamponade, esophageal injury, groin complications, vascular access complications, thromboembolic events, procedure duration, skin‐to‐skin duration, left atrial dwell time, and fluoroscopy time). Careful attention was paid to identify studies with overlapping patient populations to avoid potential bias from repeated patients. In cases where studies shared patient data from the same database and time periods, only studies with higher methodological quality and more robust analytical approaches, such as propensity score matching, were included in the pooled analysis for each outcome; otherwise, selection was based on the study with a higher sample size.

### 2.5 Risk of Bias Assessment

Two authors independently assessed the risk of bias of the included cohort and case–control studies using the Newcastle–Ottawa scale (NOS), which comprises three domains: selection process (4 points), comparability assessment between the two groups (2 points), and assessment of reported outcomes (3 points). Assessment judgments were classified as “good quality,” “fair quality,” or “poor quality.”

The risk of bias assessment of the included randomized controlled trial was conducted using the Cochrane Risk of Bias 2 tool (RoB‐2), which involves evaluating five domains: randomization process (selection bias), adherence to the intended intervention (performance bias), outcome measurement (detection bias), presence of missing outcome data (attrition bias), and selection of reported results. The authors' assessment judgments were classified as “low risk of bias,” “high risk of bias,” or “some concerns.”

For nonrandomized studies, the risk of bias in nonrandomized studies (ROBINS‐1) tool was used, which includes eight domains: confounding, selection of participants, classification of interventions, deviation from intended interventions, bias in measurement of the outcome, bias due to missing outcome data, bias in selection of the reported result, and other potential biases. The authors' assessment judgments were classified as “low risk of bias,” “moderate risk of bias,” or “serious risk of bias.” Any inconsistencies among the authors were discussed with a third party.

### 2.6 Statistical Analysis

We reconstructed individual patient data (IPD) from studies reporting Kaplan–Meier (KM) arrhythmia‐free survival curves using a validated algorithm. Survival probabilities and censoring information were extracted from published Kaplan–Meier curves using WebPlotDigitizer. Curve digitization was performed independently by two investigators, and discrepancies were resolved through discussion and consensus among the investigators. Censoring information was estimated using the numbers‐at‐risk reported in each study according to a validated reconstruction algorithm.

Reconstructed IPD was used to estimate hazard ratios (HRs) and corresponding 95% confidence intervals (CIs) using Cox proportional hazards models. The proportional hazards assumption was assessed before the model fitting. When hazard ratios were reported in the original studies, reconstructed estimates were compared with published values to assess consistency and validate the reconstruction process. For studies with zero events in one treatment arm, a continuity correction of 0.5 was applied to the data.

Dichotomous outcomes were pooled using risk ratios (RRs) with 95% confidence intervals (CIs) for outcomes with relatively high event rates (> 10%). For dichotomous outcomes with low event rates (< 10%), odds ratios (ORs) with 95% CIs were used, as ORs provide more stable estimates of rare events. Continuous outcomes were pooled using mean differences (MDs) with 95% CIs. Meta‐analyses were performed using the DerSimonian–Laird random‐effects model. Subgroup analyses were conducted according to the thermal ablation modality (radiofrequency ablation [RFA] or cryoballoon ablation [CBA]). Leave‐one‐out sensitivity analyses were performed when significant heterogeneity was present, defined as a Cochran's *Q* test *p* < 0.10 or an *I* ^2^ value ≥ 50%. Statistical analyses were conducted using STATA MP version 18 for Windows and R software (version 4.4.2).

## 3 Results

### 3.1 Literature Search

**FIGURE 1:**
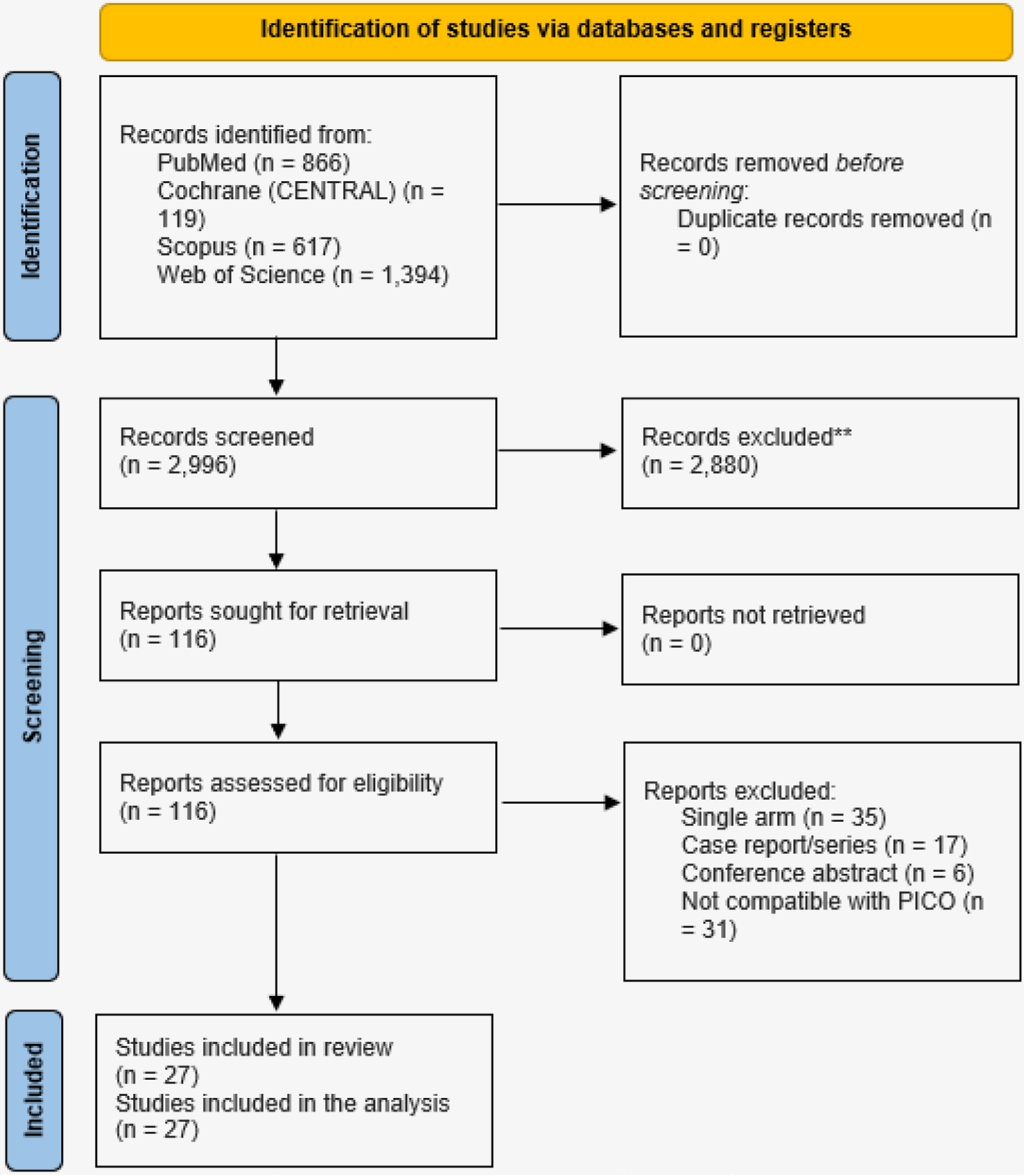
PRISMA flow diagram.

A total of 2996 articles were included. After removing duplicates and screening the titles and abstracts, 116 studies were retained for full‐text screening. A total of 27 studies met our inclusion criteria. Among these 27 studies, 23 were cohort studies [10, 11, 13, 14, 15, 16, 17, 18, 19, 20, 21, 22, 23, 24, 25, 26, 27, 28, 29, 30, 31, 32, 33], two were non‐randomized studies [34, 35], one was a randomized controlled trial [36], and one was a case–control study [37]. The PRISMA flow diagram is shown in Figure 1.

### 3.2 Characteristics of the Included Studies

Our meta‐analysis included 27 studies, comprising 8546 patients (3012 in the PFA group and 5534 in the thermal ablation group), published between 2020 and 2025, which investigated the efficacy of PFA compared to thermal ablation (CBA or RFA) in patients with AF undergoing first‐time PVI. The summary and baseline characteristics of the included studies are presented in Table 1 and Supplementary Table 2, respectively. The reported safety outcomes of each study are presented in Supplementary Table 3.

**TABLE 1:** Summary characteristics of the included studies.

Study ID	Study design	Patients	PFA technique	PFA procedure details	Thermal ablation type	Thermal ablation technique	Procedure details	Follow‐up (months)	Location	Database source
Badertscher 2023 [13]	Prospective cohort study	First‐time PVI patients with paroxysmal or persistent AF	FARAPULSE system with CARTO3	3D mapping system (CARTO3), PFA system used for PV isolation	CBA	PolarX cryoballoon system with spiral mapping catheter	Cryoablation, fluoroscopy guidance	13	University Hospital Basel, Switzerland	SWISS‐AF‐PVI registry (NCT03718364)
Cochet 2021 [16]	Prospective cohort study	First‐time PVI patients with paroxysmal AF	Farapulse (12‐F over‐the‐wire catheter)	Repeated 8 applications per vein, repositioning/rotation for complete PVI	RFA and CBA	RFA (Thermocool Smarttouch) or CBA (Arctic Front Advance)	RFA or CBA with specific settings for power, duration, and contact force	3	Bordeaux University Hospital, France	IMPULSE (NCT03700385), PEFCAT (NCT03714178)
Maurhofer 2023 [10]	Prospective cohort study	First‐time PVI patients with paroxysmal AF	Farapulse (12‐F Farawave catheter)	3D mapping (CARTO3), 32 applications per PV in basket and flower configurations	CBA and RFA	CBA (Arctic Front), RFA (Thermocool Smarttouch)	Cryoballoon at PV ostia, 2‐min freezes; RFA: CARTO3 for precise targeting	12	Inselspital, Bern University Hospital, Switzerland	SWISS‐AF‐PVI Database
Rattka 2024 [37]	Case–control study	First PVI patients with paroxysmal or persistent AF	FARAWAVE, basket and flower configurations	8 applications/PV, bidirectional block confirmed	CBA	POLARx, cryoballoon ablation	180 s freeze, PV occlusion confirmed with contrast	12	Essen University Medical Center, Germany	Institutional records
Della Rocca 2023 [11]	Retrospective review	First‐time PVI patients with nonvalvular paroxysmal AF	Farawave PFA system	4 pairs of applications per PV, repositioning every 36°	CBA and RFA	CBA: POLARx; RFA: Thermocool SmartTouch	CBA: 240 s freeze, PV occlusion confirmed; RFA: WACA ablation, 45 W RF power	12	Multiple centers across Europe and the US	Individual prospective registries
Schipper 2023 [20]	Retrospective cohort study	First‐time PVI patients with paroxysmal or persistent AF	FARAWAVE PFA system, basket and flower configurations	4 impulses/PV; optional 3D voltage mapping; entrance block confirmed with FARAWAVE	CBA	POLARx Cryoballoon system, single freeze per PV	Freeze based on TTI or nadir temperature; PV occlusion with contrast; entrance block confirmed with POLARMAP	9	University of Cologne, Germany	Electronic Data Capture System (REDCap Database)
Urbanek 2023 [21]	Retrospective cohort study	First‐time PVI patients with paroxysmal or persistent AF	FARAWAVE PFA system	8 impulses/PV, entrance block confirmed	CBA	Arctic Front Advance CB2	Single freeze/PV (240 s); terminated if esophageal temperature < 15°C	12	Cardiologisches Centrum Bethanien, Germany	Institutional Registry Database
Van de kar 2023 [22]	Retrospective cohort study	First PVI patients with atrial fibrillation	FARAWAVE system	8 applications/PV, entrance and exit block confirmed	CBA	Arctic Front Cryoballoon	Freeze terminated if esophageal temp < −40°C	6	Catharina Hospital Heart Centre, Netherlands	Institutional registry
Wahedi 2024 [23]	Prospective single‐center study	First‐time PVI patients with paroxysmal or persistent AF	Pentaspline PFA catheter	8 applications per PV, 3D mapping of the LA	CBA	27‐mm Arctic Front catheter	Freezing for 180–240 s depending on operator	NA	Hamburg, Germany	Electronic medical records
Chaumont 2024 [15]	Prospective single‐center study	First‐time PVI patients with paroxysmal or persistent AF	Pentaspline catheter	8 applications per PV in basket and flower configurations	CBA	28‐mm Arctic Front Advance	Cryoablation duration: 240 s, 180 s for right superior PV	12	Rouen University Hospital, France	Institutional database
Kueffer 2024 [17]	Prospective Cohort Study	First‐time PVI patients with persistent AF	Pulsed‐field ablation (Farapulse system)	Biphasic pulse applications, 4 per PV	CBA and RFA	CBA: Arctic Front Advance; RFA: SmartTouch SF	CBA: 28‐mm balloon, time‐to‐effect +2 min freeze strategy; RFA: CLOSE protocol	12	Inselspital, Bern University Hospital, Switzerland	SWISS‐AF‐PVI Database
Jungen 2024 [24]	Retrospective registry study	Overweight and obese patients with symptomatic AF	Biphasic pulse PFA with FARAPULSE system	8 applications per PV, circumferential PV isolation confirmed	CBA	POLARx cryoballoon	180‐s freezes per PV, phrenic nerve pacing during right‐sided PV ablation	12	University Hospital Essen, Germany	Institutional registry database
Calvert 2024 [25]	Retrospective single‐center study	First‐time catheter ablation patients for AF	Biphasic waveform applications	PVI with FARAPULSE catheter, performed under general anesthesia	CBA and RFA	CB: Arctic Front Advance; RF: ThermoCool SmartTouch	CB: Cryoablation with PV isolation confirmed; RF: PVI with CARTO mapping	12	Liverpool Heart and Chest Hospital, UK	Institution's registry
Katov 2024 [26]	Prospective, single‐center study	First‐time PVI patients with symptomatic AF	Pulsed Field Ablation using Farawave	8 energy applications per PV	CBA	PolarX Fit balloon	180 s of freezing per PV	NA	Ulm University Heart Center, Germany	ATRIUM registry (DRKS00013013)
My 2023 [18]	Prospective single‐center study	First‐time PVI patients with symptomatic AF	Pulsed‐field ablation (Pentaspline Farawave catheter)	PVI with 8 pulses/PV at 2.0 kV in “basket” and “flower” configurations	RFA	Compliant RF balloon ablation	PVI with compliant RF balloon; 15 W, 60s per electrode	NA	Hamburg, Germany	Prospective single‐center observational study database (NCT05521451)
Osmancik 2023 [36]	Prospective, randomized study	Patients with symptomatic AF undergoing first ablation	Pentaspline catheter, biphasic waveform	4 basket +4 flower applications/PV; optional posterior wall, mitral isthmus ablations	RFA	CARTO 3 mapping, ablation index guided	CARTO 3 system, ablation index 400–450 anterior, 350–400 posterior PVs	NA	University Hospital Kralovske Vinohrady, Czech Republic	ClinicalTrials.gov (NCT05603637)
Popa 2023 [19]	Retrospective, single‐center study	First‐time PVI patients with symptomatic AF	Biphasic pulsed field ablation	Farawave catheter, 4 basket +4 flower pulses per PV, entrance block confirmed	RFA	Standard RFA (30–40 W), HPSD‐70 W (70 W/5–7 s), HPSD‐90 W (90 W/4 s)	Wide antral circumferential PVI, point‐by‐point lesions, PVI confirmed by entrance block and adenosine challenge	6	Munich, Germany	Prospective, computerized database
De Becker 2024 [14]	Retrospective analysis	First‐time PVI patients with symptomatic AF	High‐voltage biphasic pulses	Biphasic pulses (8/circle; basket and flower) with Farawave catheter	RFA	Point‐by‐point ablation, 90 W/4 s or 35‐50 W	CARTO‐3 system, nMARQ generator, CLOSE protocol with QDOT catheter	6	Multiple centers across Europe	PFA Group: AZ Sint Jan Hospital registry; RF Group: PowerPlus study database
Osmancik 2024 [34]	Prospective, non‐randomized study	Symptomatic AF, first‐ever ablation patients	Pulsed‐field ablation (Farawave catheter)	PFA procedure involved sinus rhythm restoration after ablation	RFA	High‐power short‐duration radiofrequency (HPSD‐RF)	Point‐by‐point PVI, 3.5 mm irrigated‐tip catheter, 90 W/4 s for PVI	NA	University Hospital Královské Vinohrady, Czech Republic	ClinicalTrials.gov (NCT06096428)
Reinsch 2024 [35]	Retrospective, non‐randomized study	First‐time PVI patients with paroxysmal AF	Pulsed‐field ablation using Farawave catheter	8 applications per PV (4 basket, 4 flower), additional ablation for persistent AF	HPSD‐RF	High‐power short‐duration (HPSD) radiofrequency ablation	Point‐by‐point PVI, 45 W power, saline irrigation, ablation index (AI) 450 posterior/inferior/roof	12	Alfried Krupp Hospital, Essen, Germany	Institutional electronic registry database
Popa 2024 [27]	Observational, prospective study	Patients with paroxysmal or nonparoxysmal AF	FaraDrive sheath, Farawave catheter	Multiple PFA deliveries in basket and flower configurations	RFA	High‐density electroanatomical mapping	Power‐controlled or temperature‐controlled mode for ablation	NA	4 high‐volume European centers	Computerized database of the four high‐volume European centers
Soubh 2024 [29]	Retrospective analysis	First‐time PVI patients with symptomatic AF	Steerable PFA sheath (Faradrive)	3D mapping, 8 pulses per PV (4 “basket” + 4 “flower” configurations)	vHPSD‐RF	High‐density anatomical and voltage mapping	3D mapping, circumferential lesions with QDOT MICRO catheter, 90 W for 4 s	6	University Medical Center Göttingen, Germany	Medical records of patients who underwent PVI at the center
Wormann 2023 [28]	Retrospective analysis	Patients with symptomatic AF undergoing first ablation	FARAPULSE system	PFA catheter positioned at the PV antrum, delivering PFA impulses (8 per PV antrum)	VHPSD‐PVI	Very high power short duration ablation (VHPSD)	Circumferential PVI using an electroanatomic mapping system	12	Köln, Germany	Electronic case report form (CRF) using the RedCap Database
Pannone 2024 [30]	Retrospective observational study	Patients with persistent AF undergoing first ablation	Farapulse PFA system	4 applications in basket and 4 in flower configuration per PV	CBA	POLARx or Arctic Front Pro	Cryoenergy applications lasting 180 s, targeting −40°C within the first 60 s	12	Brussel, Belgium	Electronic medical records (EMR)
Nakasone 2025 [31]	Retrospective observational study	Patients with paroxysmal, persistent, or long‐standing persistent AF	Farapulse PFA system	At least four pairs of PFA applications per vein	CBA and RFA	CBA: Arctic Front Advance Pro; RFA: Carto 3	CBA: 28‐mm cryoballoon catheter; RFA: Open‐irrigated RF ablation catheter	12	Six centers across Europe and the US	Multiple institutional registries
Wang 2024 [32]	Prospective study	Patients with paroxysmal AF undergoing first‐time ablation	LEAD‐PFA system	8F alternating current biphasic pulse catheter	CBA and RFA	CBA: Nitron CryoConsole; RFA: CARTO 3 System	CBA: 15‐F steerable sheath; RFA: 20‐pole circumferential catheter	24	Renmin Hospital of Wuhan University, China	Department of Cardiology
Zhang 2025 [33]	Retrospective observational study	Patients with paroxysmal non‐valvular AF and heart failure	Dedicated PFA ablation generator	PVI guided by the 3D model, 8–12 ablation cycles per PV	CBA and RFA	CBA: Cryoballoon and Achieve mapping catheters; RFA: CARTO or Ensite 3D mapping system	CBA: Cryoballoon advanced into PVs; RFA: Open irrigated tip catheter with contact force monitoring	12	Second Xiangya Hospital, China	Cardiology Department

### 3.3 Risk of Bias Assessment

The Newcastle–Ottawa Scale (NOS) was used for cohort studies. In 23 studies, the patients were accurately represented, the exposure was verified through secure records, and the outcome of interest was not present at the start of the study. The comparative group was not from the same community in two studies [14, 16]. The study groups were not comparable in four studies [13, 24, 29, 32]. All studies had a sufficient follow‐up period for their intended outcomes to occur without significant loss to follow‐up, as shown in Table 2. NOS was used for the single included case–control study, which showed a low risk of bias (Supplementary Table 4).

**TABLE 2:** NOS for the included cohort studies.

Study ID	Selection (maximum 4)				Outcomes selected	Comparability (maximum 2)	Outcome (maximum 3)			Total score (maximum 9)	Overall appraisal (poor, fair, or good)
	1	2	3	4	5		6	7	8		
	Representativeness of exposed cohort	Selection of nonexposed cohort	Ascertainment of exposure	Demonstration that outcome of interest was not present at start of study	Comparability of cohorts on the basis of the design or analysis		Assessment of outcome	Was follow‐up long enough for outcomes to occur	Adequacy of follow up of cohorts		
	(0 or ✵)	(0 or ✵)	(0 or ✵)	(0 or ✵)	(0, ✵, or ✵✵)		(0 or ✵)	(0 or ✵)	(0 or ✵)		
Badertscher 2023	✵	✵	✵	✵	Age, Comorbidities	0	✵	✵	✵	7	Good
De Becker 2024	✵	0	✵	✵	PSM study (Age, Comorbidities)	✵✵	✵	✵	✵	8	Good
Chaumont 2024	✵	✵	✵	✵	PSM study (Age, Comorbidities)	✵✵	✵	✵	✵	9	Good
Cochet 2021	✵	0	✵	✵	Age, Comorbidities	✵	✵	✵	✵	7	Good
Kueffer 2024	✵	✵	✵	✵	Age, Comorbidities	✵	✵	✵	✵	8	Good
Maurhofer 2023	✵	✵	✵	✵	PSM study (Age, Comorbidities)	✵✵	✵	✵	✵	9	Good
My 2023	✵	✵	✵	✵	Age, Comorbidities	✵	✵	✵	✵	8	Good
Popa 2023	✵	✵	✵	✵	Age, Comorbidities	✵	✵	✵	✵	8	Good
Della Rocca 2023	✵	✵	✵	✵	PSM study (Age, Comorbidities)	✵	✵	✵	✵	8	Good
Schipper 2023	✵	✵	✵	✵	Age, Comorbidities	✵	✵	✵	✵	8	Good
Urbanek 2023	✵	✵	✵	✵	Age, Comorbidities	✵	✵	✵	✵	8	Good
van de Kar 2023	✵	✵	✵	✵	Age, Comorbidities	✵	✵	✵	✵	8	Good
Wahedi 2024	✵	✵	✵	✵	Age, Comorbidities	✵	✵	✵	✵	8	Good
Jungen 2024	✵	✵	✵	✵	Age, Comorbidities	0	✵	✵	✵	9	Good
Calvert 2024	✵	✵	✵	✵	Age, Comorbidities	✵	✵	✵	✵	8	Good
Katov 2024	✵	✵	✵	✵	Age, Comorbidities	✵	✵	✵	✵	8	Good
Popa 2024	✵	✵	✵	✵	Age, Comorbidities	✵	✵	✵	✵	8	Good
Wormann 2023	✵	✵	✵	✵	Age, Comorbidities	✵	✵	✵	✵	8	Good
Soubh 2024	✵	✵	✵	✵	Age, Comorbidities	0	✵	✵	✵	7	Good
Pannone 2024	✵	✵	✵	✵	Age, Comorbidities	✵	✵	✵	✵	8	Good
Nakasone 2025	✵	✵	✵	✵	PSM study (Age, Comorbidities)	✵✵	✵	✵	✵	9	Good
Wang 2024	✵	✵	✵	✵	Age, Comorbidities	0	✵	✵	✵	7	Good
Zhang 2025	✵	✵	✵	✵	Age, Comorbidities	✵	✵	✵	✵	8	Good

For non‐randomized studies, the ROBINS‐1 tool was utilized, revealing a low risk of bias (Supplementary Figure 1). ROB‐2 was used for the randomized study, which showed some concerns regarding bias (Supplementary Figure 2).

Potentially overlapping cohorts were carefully assessed based on institution, registry source, and recruitment period (Supplementary Table 5) and definitions of the blanking period were generally consistent across studies and are summarized in Supplementary Table 6.

### 3.4 Outcomes

#### 3.4.1 Tachyarrhythmia Recurrence

**FIGURE 2:**
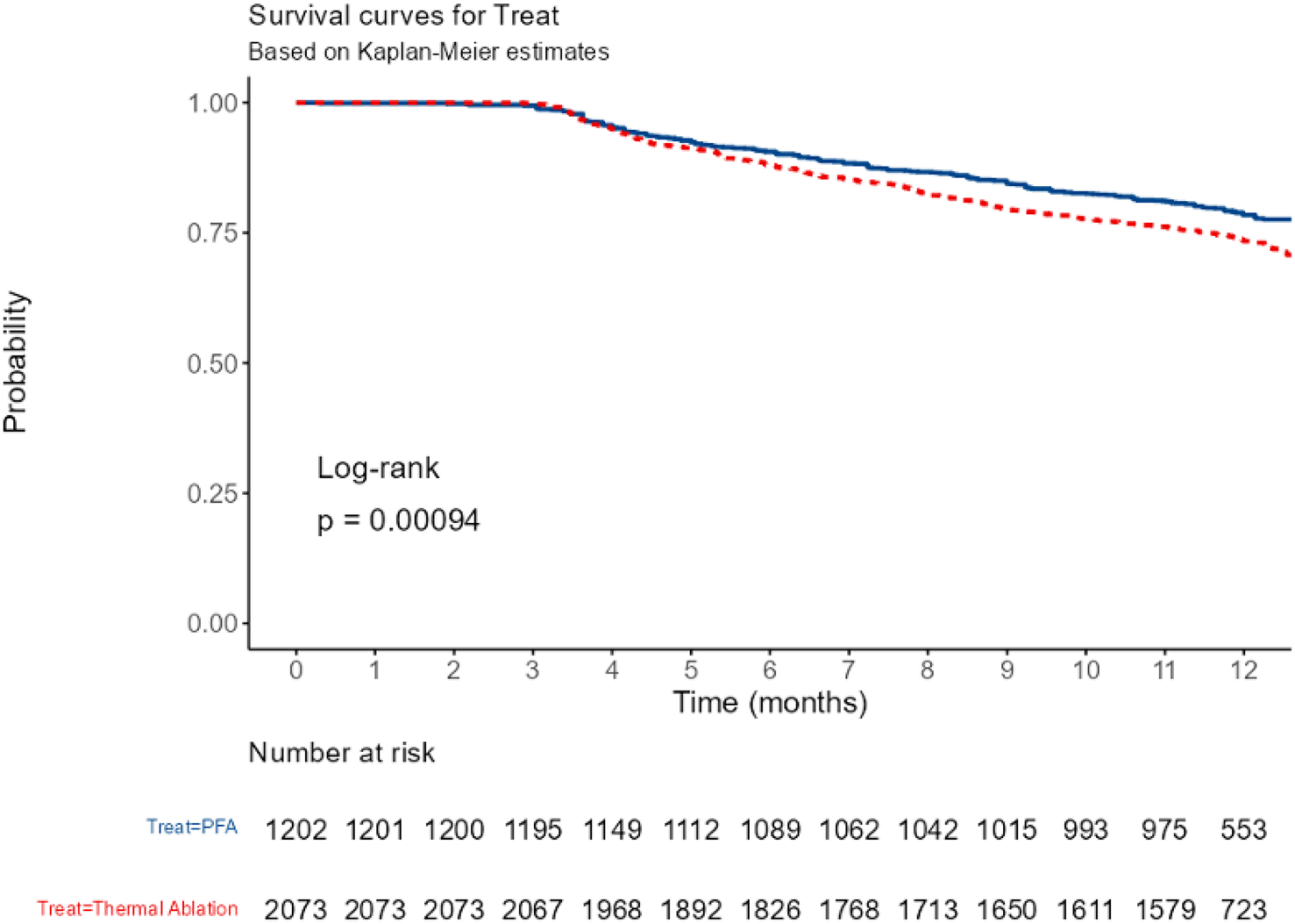
Kaplan–Meier curve estimates of freedom from tachyarrhythmia recurrence for PFA versus Thermal ablation.

A KM reconstruction was performed using data from ten studies and revealed that the recurrence free survival rate at 12 months was 78.4% (95% CI: 76.1%–80.8%) in the PFA group and 73.4% (95% CI: 71.5%–75.4%) in the thermal ablation group, with a significantly higher risk of tachyarrhythmia recurrence in the thermal ablation group compared to PFA (HR: 1.28, 95% CI [1.10, 1.48], *p* = 0.001), as shown in Figure 2.

**FIGURE 3:**
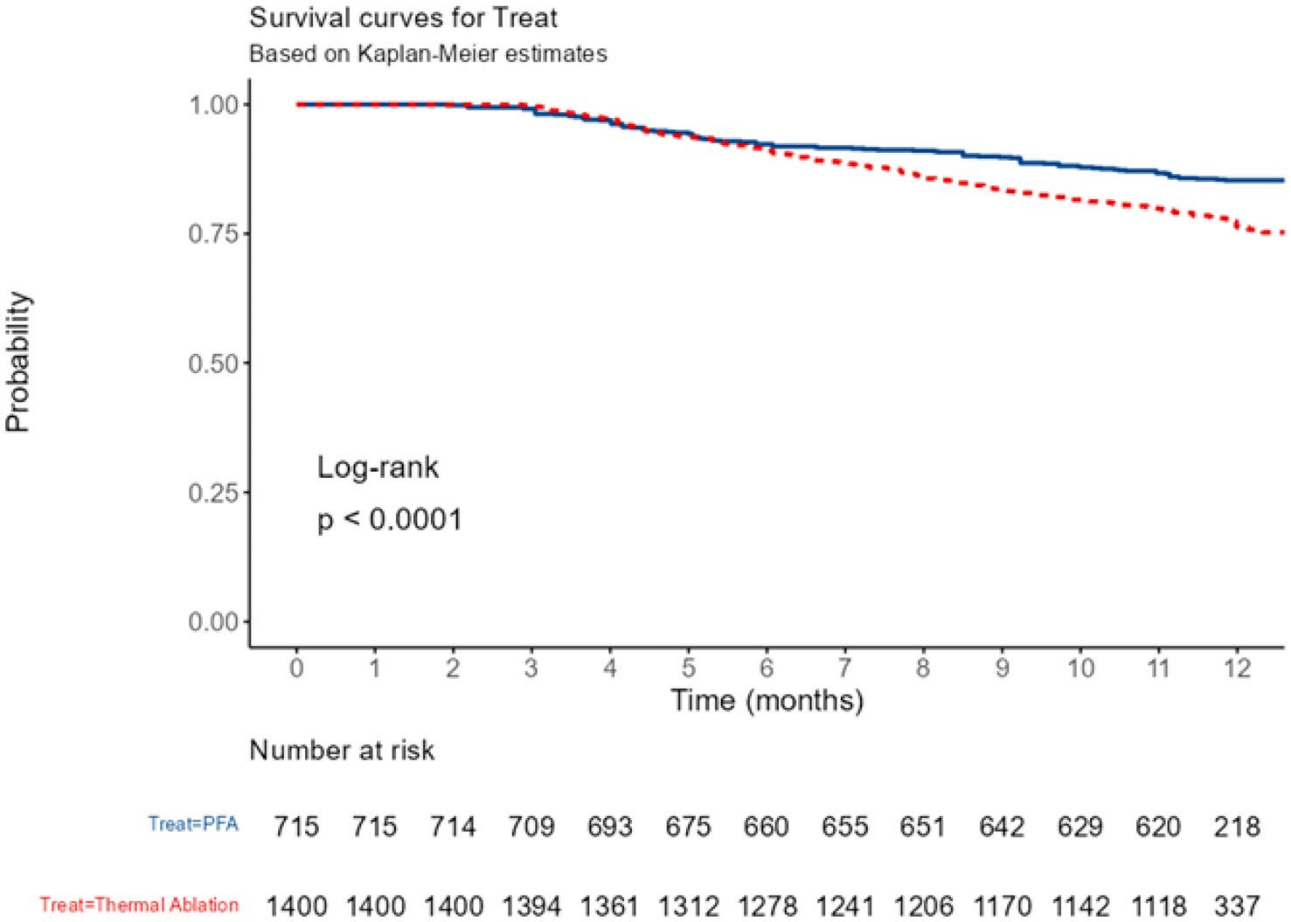
Kaplan–Meier curve estimates of freedom from tachyarrhythmia recurrence for PFA versus thermal ablation in paroxysmal AF patients.

For patients with paroxysmal AF, PFA was significantly associated with a lower risk of arrhythmia recurrence than thermal ablation (HR: 1.63, 95% CI [1.31, 2.03], *p* < 0.001), as shown in Figure 3. There was no significant difference between PFA and thermal ablation in patients with persistent AF (HR: 1.13, 95% CI [0.92, 1.41], *p* = 0.25), as shown in Supplementary Figure 3.

A subgroup analysis was performed based on the thermal ablation type, which showed that PFA was significantly associated with a lower risk of tachyarrhythmia recurrence than CBA (HR: 1.23, 95% CI [1.04, 1.45], *p* = 0.015) and RFA (HR: 1.33, 95% CI [1.13, 1.58], *p* = 0.001), as shown in Supplementary Figure 4.

#### 3.4.2 Atrial Fibrillation Recurrence

**FIGURE 4:**
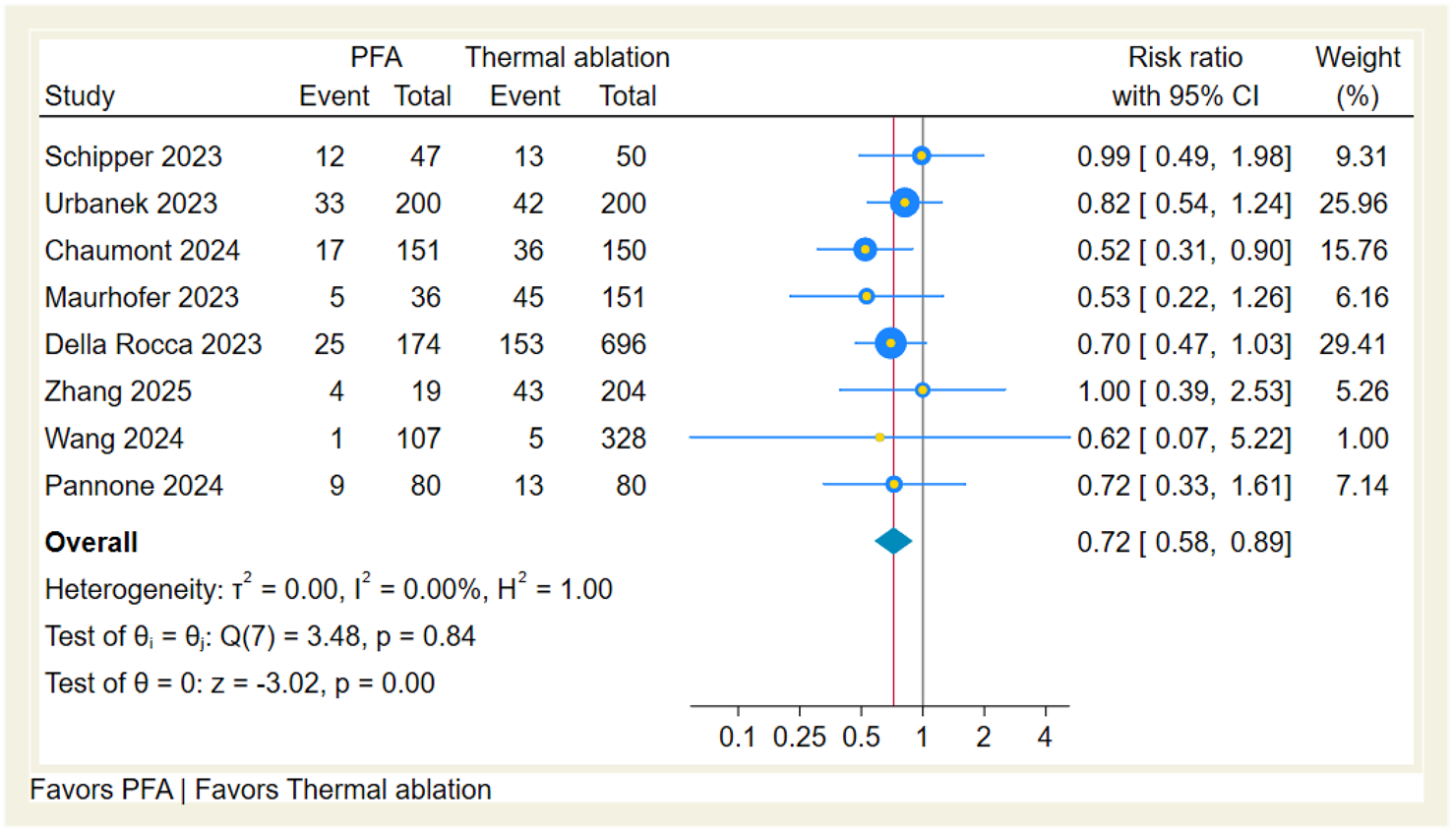
Forest plot of AF recurrence for PFA versus thermal ablation.

Seven studies assessed the incidence of AF recurrence, which showed that PFA was significantly associated with lower AF recurrence than thermal ablation after 1 year of follow‐up (RR = 0.72, 95% CI [0.58, 0.89], *p* < 0.001). The pooled studies were homogeneous (*I* ^2^ = 0%, *p* = 0.84), as shown in Figure 4.

A subgroup analysis was conducted according to thermal ablation type which preferred PFA over both: CBA (RR = 0.78, 95% CI [0.65, 0.93], *p* = 0.01) and RFA (RR = 0.76, 95% CI [0.60, 0.96], *p* = 0.02), as shown in Supplementary Figure 5.

A L' Abbe plot was conducted to assess the study‐specific event rate between the two groups and to explore the heterogeneity between studies, which showed that six studies were below the reference line, indicating higher AF recurrence in the thermal ablation group than in the PFA group, and two studies were on the reference line, indicating no difference in AF recurrence between the two groups (Supplementary Figure 6).

#### 3.4.3 Durable PVI

There was no significant difference between PFA and thermal ablation in terms of durable PVI events (RR = 0.96, 95% CI [0.87, 1.06], *p* = 0.46). The pooled studies were homogeneous (*I* ^2^ = 0%, *p* = 0.95), as shown in Supplementary Figure 7. We conducted a subgroup analysis based on the thermal ablation type, which revealed no significant difference between the two groups according to CBA (RR = 1.00, 95% CI [0.89, 1.12], *p* = 0.94) or RFA (RR = 0.92, 95% CI [0.82, 1.04], *p* = 0.21) (Supplementary Figure 8).

#### 3.4.4 Redo Procedure

There was no significant difference between PFA and thermal ablation regarding redo procedures (RR = 0.85, 95% CI [0.67, 1.07], *p* = 0.17). The pooled studies were heterogeneous (*I* ^2^ = 50%, *p* = 0.03), as shown in Supplementary Figure 9. A leave‐one‐out sensitivity analysis was conducted, and no single study had a disproportionate effect on the overall pooled analysis, as shown in Supplementary Figure 10. A subgroup analysis was conducted based on thermal ablation type and showed no significant difference between the two groups: CBA (RR = 0.87, 95% CI [0.67, 1.12], *p* = 0.28) and RFA (RR = 0.80, 95% CI [0.60, 1.08], *p* = 0.14), as shown in Supplementary Figure 11.

### 3.5 Safety Outcomes

#### 3.5.1 Transient PNP

PFA was associated with fewer patients with transient PNP than thermal ablation (OR = 0.23, 95% CI [0.11, 0.49], *p* < 0.001). The pooled studies were homogeneous (*I* ^2^ = 0%; *p* = 0.96), as shown in Supplementary Figure 12. In the subgroup analysis based on thermal ablation type, PFA was associated with fewer patients with transient PNP than CBA (OR = 0.17, 95% CI [0.08, 0.36], *p* < 0.001), while there was no significant difference between PFA and RFA (OR = 1.04, 95% CI [0.25, 4.31], *p* = 0.96), as shown in Supplementary Figure 13.

#### 3.5.2 Persistent PNP

There was no significant difference between PFA and thermal ablation in terms of persistent PNP (OR = 0.45, 95% CI [0.17, 1.16], *p* = 0.10). The pooled studies were homogeneous (*I* ^2^ = 0%; *p* = 0.96), as shown in Supplementary Figure 14. Our subgroup analysis according to thermal ablation type favored PFA over CBA (OR = 0.31, 95% CI [0.11, 0.82], *p* = 0.02), while PFA showed no significant difference compared with RFA (OR = 0.91, 95% CI [0.24, 3.43], *p* = 0.89), as shown in Supplementary Figure 15.

#### 3.5.3 Cardiac Tamponade

The pooled OR did not show a significant difference between PFA and thermal ablation regarding cardiac tamponade (OR = 1.69, 95% CI [0.89, 3.22], *p* = 0.11). The pooled studies were homogeneous (*I* ^2^ = 0%; *p* = 0.95), as shown in Supplementary Figure 16. Our subgroup analysis according to thermal ablation type favored CBA over PFA (OR = 2.39, 95% CI [1.08, 5.31], *p* = 0.03), while there was no significant difference between PFA and RFA (OR = 1.15, 95% CI [0.51, 2.59], *p* = 0.75), as shown in Supplementary Figure 17.

#### 3.5.4 Esophageal Injury

There was no significant difference between PFA and thermal ablation in terms of esophageal injury (OR = 0.77, 95% CI [0.26, 2.32], *p* = 0.65). The pooled studies were homogeneous (*I* ^2^ = 0%; *p* = 0.80), as shown in Supplementary Figure 18. The subgroup analysis showed no significant difference between the two groups according to CBA (OR = 0.57, 95% CI [0.16, 2.09], *p* = 0.40) or RFA (OR = 0.63, 95% CI [0.17, 2.30], *p* = 0.49), as shown in Supplementary Figure 19.

#### 3.5.5 Groin Complications

The pooled analysis showed no significant difference between PFA and thermal ablation (OR = 0.99, 95% CI [0.48, 2.01], *p* = 0.97). The pooled studies were homogeneous (*I* ^2^ = 11.9%; *p* = 0.34), as shown in Supplementary Figure 20. According to the subgroup analysis based on thermal ablation type, no significant difference was observed between the two groups: CBA (OR = 0.81, 95% CI [0.34, 1.96], *p* = 0.64) and RFA (OR = 1.07, 95% CI [0.38, 3.03], *p* = 0.89), as shown in Supplementary Figure 21.

#### 3.5.6 Vascular Access Complications

There was no significant difference between PFA and thermal ablation regarding vascular access complications (OR = 0.84, 95% [CI 0.40, 1.74], *p* = 0.63). The pooled studies were homogeneous (*I* ^2^ = 0%; *p* = 0.97), as shown in Supplementary Figure 22. According to the subgroup analysis based on thermal ablation type, there was no significant difference between the two groups regarding CBA (OR = 0.85, 95% [CI 0.36, 2.02], *p* = 0.72) or RFA (OR = 0.82, 95% [CI 0.27, 2.49], *p* = 0.72), as shown in Supplementary Figure 23.

#### 3.5.7 Thromboembolic Events

There was no significant difference between PFA and thermal ablation regarding thromboembolic events (OR = 1.31, 95% CI [0.64, 2.67], *p* = 0.46). The pooled studies were homogeneous (*I* ^2^ = 0%; *p* = 0.99), as shown in Supplementary Figure 24. Our subgroup analysis did not favor either of the two studied groups regarding CBA (OR = 1.08, 95% CI [0.44, 2.66], *p* = 0.87) or RFA (OR = 1.35, 95% CI [0.54, 3.34], *p* = 0.52), as shown in Supplementary Figure 25.

### 3.6 Procedural Metrics

#### 3.6.1 Procedure Duration

PFA was significantly associated with a shorter procedure duration than thermal ablation (MD = −27.30, 95% [CI −32.09, −22.52], *p* < 0.001). The pooled studies were heterogeneous (*I* ^2^ = 94.76%; *p* < 0.001), as shown in Supplementary Figure 26. A leave‐one‐out sensitivity analysis was performed and showed that no single study had a disproportionate effect on the overall pooled analysis, as shown in Supplementary Figure 27. Subgroup analysis based on thermal ablation type showed no significant difference between the two groups in terms of CBA (MD = −9.44, 95% [CI −14.16, −4.72], *p* < 0.001) and RFA (MD = −47.62, 95% [CI −58.74, −36.49], *p* < 0.001), as shown in Supplementary Figure 28.

#### 3.6.2 Skin‐To‐Skin Duration

PFA was associated with a shorter skin‐to‐skin duration than thermal ablation (MD = −33.11, 95% [CI −45.23, −20.98], *p* < 0.001). The pooled studies were heterogeneous (*I* ^2^ = 91.32%; *p* < 0.001), as shown in Supplementary Figure 29. A leave‐one‐out sensitivity analysis was conducted and showed that no single study had a disproportionate effect on the overall pooled analysis, as shown in Supplementary Figure 30. Our subgroup analysis according to thermal ablation type preferred PFA over both: CBA (MD = −13.55, 95% [CI −22.49, −4.61], *p* < 0.001) and RFA (MD = −37.84, 95% [CI −51.49, −24.19], *p* < 0.001), as shown in Supplementary Figure 31.

#### 3.6.3 LA Dwell Time

PFA was associated with a lower LA dwell time than thermal ablation (MD = −32.04, 95% [CI −43.67, −20.42], *p* < 0.001). The pooled studies were heterogeneous (*I* ^2^ = 97.26%; *p* < 0.001), as shown in Supplementary Figure 32. We conducted a leave‐one‐out sensitivity analysis and showed that no single study had a disproportionate effect on the overall pooled analysis, as shown in Supplementary Figure 33. Subgroup analysis showed that PFA was associated with a lower LA dwell time than CBA (MD = −9.37, 95% [CI −15.64, −3.10], *p* < 0.001) and RFA (MD = −44.44, 95% [CI −56.60, −32.27], *p* < 0.001), as shown in Supplementary Figure 34.

#### 3.6.4 Fluoroscopy Time

Thermal ablation was associated with a lower fluoroscopy time than PFA (MD = 2.98, 95% [CI 0.70, 5.25], *p* = 0.01). The pooled studies were not homogeneous (*I* ^2^ = 98.47%; *p* < 0.001), as shown in Supplementary Figure 35. A leave‐one‐out sensitivity analysis was conducted and showed that no single study had a disproportionate effect on the overall pooled analysis, as shown in Supplementary Figure 36. A subgroup analysis based on ablation type showed no significant difference between PFA compared to CBA (MD = 0.21, 95% [CI −1.68, 2.11], *p* = 0.82). RFA was associated with a significantly lower fluoroscopy time than PFA (MD = 6.23, 95% [CI 2.98, 9.48], *p* < 0.001), as shown in Supplementary Figure 37.

## 4 Discussion

This meta‐analysis comprehensively evaluated the efficacy and safety of PFA versus thermal ablation in patients with AF undergoing PVI for the first time. It is the largest analysis to date, combining data from 27 studies, involving a total of 8546 patients.

Our analysis found that PFA was significantly associated with a lower risk of atrial tachyarrhythmia recurrence than thermal ablation. We additionally conducted a separate analysis for patients with paroxysmal and persistent AF. PFA was favored over thermal ablation because it was associated with a lower risk of tachyarrhythmia recurrence in patients with paroxysmal AF and there was no significant difference between the two groups in patients with persistent AF. The absence of a meaningful benefit in persistent AF might be due to insufficient statistical power and smaller sample sizes. Another possibility is that persistent AF is more driven by atrial substrate remodeling and non‐PV triggers, which might decrease the relative impact of enhanced pulmonary vein isolation alone. In a further subgroup analysis comparing the types of thermal ablation (CBA and RFA), PFA was associated with lower recurrence of tachyarrhythmia than CBA and RFA. PFA was also preferred for AF recurrence over thermal ablation.

PFA had a significantly lower incidence of transient PNP than thermal ablation. According to our subgroup analysis, significantly fewer patients with transient PNP were observed in the PFA group than in the CBA group. The incidence of persistent PNP was significantly higher in the CBA group than in the PFA group. Further subgroup analysis showed that the risk of cardiac tamponade was significantly lower with CBA in comparison with PFA. While the overall analysis did not show a significant difference, this subgroup finding suggests that procedural characteristics specific to PFA systems or operator learning curves may influence the risk of tamponade. Thus, this potential safety signal should be interpreted carefully and evaluated further in future studies.

Although durable pulmonary vein isolation (PVI) rates were comparable between PFA and thermal ablation, PFA was associated with a lower rate of clinical arrhythmia recurrence. This discrepancy should be interpreted cautiously because substantial methodological heterogeneity existed across the included studies. Rhythm‐monitoring protocols varied considerably, ranging from intermittent ECG assessments combined with 24‐h, 5‐day, or 7‐day Holter monitoring to continuous surveillance using implantable cardiac devices, while some studies performed additional monitoring in symptomatic patients [11, 13, 14]. Follow‐up schedules, blanking periods, and definitions of arrhythmia recurrence differed, and this may have affected the sensitivity for detecting recurrent atrial arrhythmias and partially explain the apparent discrepancy between similar durable PVI rates and lower observed clinical recurrence after PFA. Moreover, the predominance of observational studies increases the potential for differences in patient selection and surveillance intensity, limiting causal inference. However, mechanisms other than durable pulmonary vein isolation may contribute to long‐term rhythm outcomes. While the rates of durable PVI are similar, differences in lesion quality and continuity, effects on atrial substrate, inflammatory response, autonomic modulation, and post‐ablation atrial remodeling may contribute to the recurrence of arrhythmia. However, these mechanisms are not well understood and require further study.

All forms of thermal energy, including radiofrequency and cryotherapy, tend to ablate all tissues without discrimination. There is a trade‐off between safety and efficacy; greater efficacy requires more extensive ablation, which compromises safety, and vice versa. Conversely, PFA is a non‐thermal technique that uses ultra‐fast electrical fields (less than 1 s) to target tissue. This technique destabilizes cell membranes through the formation of irreversible nanoscale pores, leading to cell death by leakage of cell contents [38, 39, 40, 41]. Importantly, different tissues have unique threshold field strengths that trigger necrosis [42, 43, 44].

PFA is particularly suitable for cardiac ablation because cardiomyocytes have some of the lowest threshold values of any tissue [44]. The most important novelty is the application of PFA technology. This energy source is based on the direct electrical effect on tissue damage with negligible thermal and barotraumatic effects, leading to a faster and safer ablation treatment [45].

Atrial tachyarrhythmia incidence was lower with PFA than with thermal ablation. PFA creates large lesions with high‐intensity electric fields without significant thermal injury and therefore minimizes the risk of gaps within the ablation lesions. However, the recurrence of thermal ablation (e.g., RFA and CBA) can occur due to incomplete isolation of PVs and fibrosis‐induced scarring that may interfere with ablation lesions. PFA uses short‐duration, high‐intensity electrical pulses to cause irreversible electroporation with a selective effect on myocytes, increasing their susceptibility to therapy. The selective effect on myocytes helps to mitigate impact on adjacent tissues, thus reducing the likelihood of collateral damage associated with AF ablation procedures [46]. These findings have important clinical implications, as the lower risk for complications with PFA may be associated with better patient outcomes and increased procedural safety. In a study conducted by Chaumont et al., no significant differences were observed between PFA and CBA in arrhythmia‐free survival after 3 months in patients with AF undergoing first PVI, while PFA was associated with a significantly higher one‐year freedom from arrhythmia recurrence rate than CBA [15]. Interestingly, Nakasone et al. [31] assessed PFA versus thermal ablation, including only elderly patients over the age of 75, and found no significant difference between the two groups.

De Campos et al. performed a meta‐analysis comparing PFA and thermal ablation in patients with AF [47]. After 1 year of follow‐up, there was no significant difference in treatment failure, defined as recurrence of atrial tachyarrhythmia, use of antiarrhythmic drugs, and cardioversion. Nevertheless, PFA was significantly associated with lower treatment failure in patients with paroxysmal AF compared to thermal ablation. These results are in agreement with ours, except for tachyarrhythmia recurrence after 1 year, which can be explained by their wider inclusion criteria (not restricted to first‐time PVI patients) and the inclusion of studies with overlapping patients that should not be combined in the same analysis, such as Badertscher et al. and Maurhofer et al. [10, 13] used the same database and overlapping timeframes; therefore, combining these two studies in a single analysis introduces bias. Additionally, the PFA group in Schipper et al. and Wormann et al. [20, 28] probably had overlapping patients, as both studies were conducted at the same institution with overlapping timeframes. In addition, a meta‐analysis by Iqbal et al. [48] showed no significant difference in PFA and thermal ablation in AF recurrence, which is probably due to the same reasons as their inclusion criteria and including overlapping patients; we avoided this issue in our analysis.

Another meta‐analysis conducted by Amin et al. [49] on patients with AF showed that PFA was significantly associated with lower phrenic nerve palsy, esophageal lesions, and AF recurrence than thermal ablation. Our findings align with theirs, except for esophageal lesions, which may be due to the lack of estimation for zero values, influencing the pooled analysis. Additionally, they did not analyze transient and persistent PNP separately. We found no significant difference between PFA and thermal ablation regarding persistent PNP, which may also be influenced by the lack of zero value estimation in their pooled analysis. Additionally, they found no significant difference between PFA and thermal ablation in atrial tachyarrhythmia recurrence, which may be because they did not exclusively include studies with first‐time PVI ablation. When Rudolph et al. [50] compared PFA and CBA in patients with AF, they found significantly lower rates of arrhythmia recurrence and phrenic nerve injuries in PFA than in CBA, while cardiac tamponade was significantly higher in PFA than in CBA. Our findings should be interpreted in the context of randomized evidence. The ADVENT trial demonstrated the noninferiority rather than superiority of PFA compared with conventional thermal ablation in patients with paroxysmal AF [7 ]. Therefore, our results should be interpreted as hypothesis‐generating and in line with current observational evidence rather than as definitive evidence of superiority.

Another concern in interpreting these findings is the heterogeneity of PFA technologies. Although our pooled analysis demonstrated favorable efficacy and safety outcomes with PFA, the included studies evaluated multiple catheter platforms with distinct designs, pulse waveforms, energy‐delivery protocols, and procedural workflows. Furthermore, most available evidence was derived from studies using the FARAPULSE/FARAWAVE platform, whereas data for newer PFA technologies remain comparatively limited. Consequently, the observed pooled estimates should not be assumed to apply equally to all currently available or future PFA systems, and device‐specific comparative studies are needed to determine whether these findings are consistent across different technologies.

Another safety issue that is emerging after PFA is coronary artery spasm. Although this complication was not uniformly reported in the included studies and could not be evaluated in our quantitative synthesis, there have been recent reports of clinically significant coronary vasospasm and ischemic events during or shortly after PFA. While coronary vasospasm was initially described when PFA was delivered adjacent to coronary arteries, more recent reports have also documented severe nitrate‐responsive coronary spasm following anatomically remote pulmonary vein isolation procedures, suggesting that this complication may not be solely attributable to direct coronary proximity [51, 52]. The underlying mechanism remains incompletely understood but may involve autonomic‐mediated vasomotor responses or electroporation‐related vascular effects. Although the currently available evidence is limited and consists primarily of registry data and case reports, these findings underscore the need for continued surveillance and standardized reporting of coronary complications in future studies to better define their incidence, mechanisms, and clinical significance.

Our systematic review and meta‐analysis provides a comprehensive synthesis of the current evidence, including 27 studies involving 8546 patients. The inclusion of a large patient population increased statistical power and improved the overall precision and generalizability of the findings. We also employed advanced analytical approaches, including individual patient data reconstruction as well as subgroup and sensitivity analyses.

Several limitations should be acknowledged. Most included studies were observational and therefore susceptible to residual confounding, selection bias, and unmeasured differences between treatment groups despite adjustment methods. We could not evaluate arrhythmia recurrence at shorter follow‐up intervals (3 and 6 months) because of insufficient available data. Variability in PFA catheter platforms, operator experience, monitoring strategies, and follow‐up duration may have influenced the observed outcomes. Procedural outcomes demonstrated substantial heterogeneity, likely reflecting differences in operator experience, institutional workflows, learning‐curve effects, mapping systems, sedation strategies, and device‐specific characteristics. Consequently, pooled estimates of procedural metrics should be interpreted with caution. Definitions of arrhythmia recurrence, rhythm‐monitoring strategies, follow‐up schedules, and blanking periods were not standardized across the included studies. Rhythm surveillance ranged from intermittent ECG assessments and Holter monitoring of varying durations to continuous monitoring with implantable cardiac devices, with some studies also incorporating symptom‐driven evaluations. Because more intensive monitoring is known to increase the detection of recurrent atrial arrhythmias, these methodological differences may have influenced reported recurrence rates, contributed to between‐study heterogeneity, and affected the pooled efficacy estimates. Although reconstruction of individual patient data from published Kaplan–Meier curves is a validated methodology, minor estimation errors may occur because of digitization and incomplete reporting of censoring information in the original studies. Publication bias was not formally assessed because most efficacy and safety outcomes included a limited number of studies, reducing the reliability of conventional methods used to detect small‐study effects. Different PFA platforms and catheter designs were used across studies, and device‐specific differences in lesion formation, workflow, and operator experience may have affected both procedural and clinical outcomes. Finally, most included studies reported outcomes at approximately 12 months; therefore, the long‐term durability of PFA beyond 1 year remains uncertain and warrants further investigation.

## 5 Clinical Implications

The findings suggest that PFA is associated with lower rates of arrhythmia recurrence and transient phrenic nerve palsy than thermal ablation in patients undergoing first‐time pulmonary vein isolation. However, because the available evidence is derived predominantly from observational studies, these findings should be interpreted as associations rather than evidence of a causal treatment effect. Consequently, treatment decisions should continue to be individualized based on patient characteristics, procedural considerations, and operator experience until adequately powered randomized trials establish the comparative effectiveness and safety of different ablation technologies. Although the observed reduction in phrenic nerve palsy may be clinically relevant, the lower risk of cardiac tamponade observed with cryoballoon ablation also highlights that the relative benefits and risks of each ablation modality should be considered during procedural planning.

## 6 Conclusion

Current evidence suggests that PFA may be associated with lower rates of tachyarrhythmia recurrence, AF recurrence, and transient PNP than thermal ablation. However, these findings were primarily derived from observational data and require confirmation in additional randomized studies.

## Author Contributions

Y.R.A.: conceptualization, literature searching, data curation, formal analysis, methodology, project administration, software, supervision, validation, writing – original draft, writing – review and editing. A.A.A.: formal analysis, software, supervision, writing – review and editing. AA: supervision, methodology, software, writing – review and editing. M.N.A.Y.: risk of bias assessment, writing‐original draft, software, writing – review and editing. O.S.E.‐S.: visualization, data curation, writing – review and editing. M.M.: writing – original draft, writing – review and editing. A.R.M.: data curation, writing – review and editing. M.A.: writing – original draft, writing – review and editing. K.A.: data curation, writing – review and editing. B.H.: formal analysis, writing – review and editing. A.‐H.S.W.: visualization, writing – review and editing. M.A.: data curation, writing – review and editing. All the authors have read and approved the final content.

## Funding

The authors have nothing to report.

## Conflicts of Interest

The authors declare no conflicts of interest.

## Supporting information


**Supplementary Figure 1.** ROBINS‐I for the included nonrandomized studies.
Supplementary Figure 2. ROB‐2 for the included randomized study.
Supplementary Figure 3. Kaplan–Meier curve estimates of freedom from tachyarrhythmia recurrence in the PFA group versus thermal ablation group in persistent AF patients.
Supplementary Figure 4. Kaplan–Meier curve estimates of freedom from tachyarrhythmia recurrence for PFA versus CBA versus RFA.
Supplementary Figure 5. Forest plot of AF recurrence for PFA versus thermal ablation.
Supplementary Figure 6. Labbe plot of AF recurrence for PFA versus thermal ablation.
Supplementary Figure 7. Forest plot of durable PVI for PFA versus thermal ablation.
Supplementary Figure 8. Forest plot of durable PVI for PFA versus thermal ablation.
Supplementary Figure 9. Forest plot of redo procedure for PFA versus thermal ablation.
Supplementary Figure 10. Leave‐one‐out sensitivity analysis of redo procedure for PFA versus thermal ablation.
Supplementary Figure 11. Forest plot of redo procedure for PFA versus thermal ablation.
Supplementary Figure 12. Forest plot of transient PNP for PFA versus thermal ablation.
Supplementary Figure 13. Forest plot of transient PNP for PFA versus thermal ablation.
Supplementary Figure 14. Forest plot of persistent PNP for PFA versus thermal ablation.
Supplementary Figure 15. Forest plot of persistent PNP for PFA versus thermal ablation.
Supplementary Figure 16. Forest plot of cardiac tamponade for PFA versus thermal ablation.
Supplementary Figure 17. Forest plot of cardiac tamponade for PFA versus thermal ablation.
Supplementary Figure 18. Forest plot of esophageal injury for PFA versus thermal ablation.
Supplementary Figure 19. Forest plot of esophageal injury for PFA versus thermal ablation.
Supplementary Figure 20. Forest plot of groin complication for PFA versus thermal ablation.
Supplementary Figure 21. Forest plot of groin complications for PFA versus thermal ablation.
Supplementary Figure 22. Forest plot of VAC for PFA versus thermal ablation.
Supplementary Figure 23. Forest plot of VAC for PFA versus thermal ablation.
Supplementary Figure 24. Forest plot of thromboembolic complications for PFA versus thermal ablation.
Supplementary Figure 25. Forest plot of thromboembolic complications for PFA versus thermal ablation.
Supplementary Figure 26. Forest plot of procedure duration for PFA versus thermal ablation.
Supplementary Figure 27. Leave‐one‐out sensitivity analysis of procedure duration for PFA versus thermal ablation.
Supplementary Figure 28. Forest plot of procedure duration for PFA versus thermal ablation.
Supplementary Figure 29. Forest plot of skin‐to‐skin duration for PFA versus thermal ablation.
Supplementary Figure 30. Leave‐one‐out sensitivity analysis of skin‐to‐skin duration for PFA versus thermal ablation.
Supplementary Figure 31. Forest plot of skin‐to‐skin duration for PFA versus thermal ablation.
Supplementary Figure 32. Forest plot of LA dwell time for PFA versus thermal ablation.
Supplementary Figure 33. Leave‐one‐out sensitivity analysis of LA dwell time for PFA versus thermal ablation.
Supplementary Figure 34. Forest plot of LA dwell time for PFA versus thermal ablation.
Supplementary Figure 35. Forest plot of Fluoroscopy time for PFA versus thermal ablation.
Supplementary Figure 36. Leave‐one‐out sensitivity analysis of fluoroscopy time for PFA versus thermal ablation.
Supplementary Figure 37. Forest plot of Fluoroscopy time for PFA versus thermal ablation.
Supplementary Table 1. Search strategy for each database.
Supplementary Table 2. Baseline characteristics of the included studies.
Supplementary Table 3. Periprocedural complications reported in the included studies.
Supplementary Table 4. NOS for the included case–control studies.
Supplementary Table 5. Assessment of potentially overlapping cohorts.
Supplementary Table 6. Blanking period of the included studies.

## Data Availability

All data generated or analyzed during this study are included in this published article.
