# Supplementary material for: Pulsed Field Ablation Versus Thermal Ablation for First‐Time Pulmonary Vein Isolation in Atrial Fibrillation: A Systematic Review and Meta‐Analysis
Source: J Arrhythm. 2026 Aug 6;42(4):e70438. doi: 10.1002/joa3.70438 (PMC13448160; doi:10.1002/joa3.70438)
Supplement: Supplementary file 1 [file JOA3-42-e70438-s001.docx]

**Supplementary table 1. Search strategy for each database.**

| **Database** | **Search Strategy** | **Articles, n** |
| --- | --- | --- |
| **Pubmed** | ("Pulsed field ablation" or "Pulsed-field ablation" or PFA or PulseSelect or Medtronic) and ("Atrial Fibrillation" or "Atrial Fibrillations" or Fibrillation or Atrial or "Auricular Fibrillation" or Auricular or "Persistent Atrial Fibrillation" or "Atrial Fibrillation" or "Persistent Atrial" or "Familial Atrial Fibrillation" or "Paroxysmal Atrial Fibrillation" or "Atrial Fibrillation" or "Paroxysmal Atrial" or "Supraventricular Tachycardia" or Supraventricular) and ("thermal ablation" or thermal or "Heat therapy" or "Heat ablation" or "Heat-based ablation" or Thermotherapy or "Radiofrequency ablation" or RF or "Radiofrequency catheter ablation" or Radiofrequency or "Catheter-based radiofrequency therapy" or "Cryotherapy ablation" or Cryotherapy or Cryo-based or Cryoablation or Cryoenergy or Cryoballoon or Cryocatheter) | 866 Articles |
| **Scopus** | TITLE-ABS-KEY(("Pulsed field ablation" OR "Pulsed-field ablation" OR PFA OR PulseSelect OR Medtronic) AND ("Atrial Fibrillation" OR "Atrial Fibrillations" OR Fibrillation OR Atrial OR "Auricular Fibrillation" OR Auricular OR "Persistent Atrial Fibrillation" OR "Atrial Fibrillation" OR "Persistent Atrial" OR "Familial Atrial Fibrillation" OR "Paroxysmal Atrial Fibrillation" OR "Atrial Fibrillation" OR "Paroxysmal Atrial" OR "Supraventricular Tachycardia" OR Supraventricular) AND ("thermal ablation" OR thermal OR "Heat therapy" OR "Heat ablation" OR "Heat-based ablation" OR Thermotherapy OR "Radiofrequency ablation" OR RF OR "Radiofrequency catheter ablation" OR Radiofrequency OR "Catheter-based radiofrequency therapy" OR "Cryotherapy ablation" OR Cryotherapy OR Cryo-based OR Cryoablation OR Cryoenergy OR Cryoballoon OR Cryocatheter)) | 617 Articles |
| **Web of Science** | TS=("Pulsed field ablation" OR "Pulsed-field ablation" OR PFA OR PulseSelect OR Medtronic) AND TS=("Atrial Fibrillation" OR "Atrial Fibrillations" OR Fibrillation OR Atrial OR "Auricular Fibrillation" OR Auricular OR "Persistent Atrial Fibrillation" OR "Atrial Fibrillation" OR "Persistent Atrial" OR "Familial Atrial Fibrillation" OR "Paroxysmal Atrial Fibrillation" OR "Atrial Fibrillation" OR "Paroxysmal Atrial" OR "Supraventricular Tachycardia" OR Supraventricular) AND TS=("thermal ablation" OR thermal OR "Heat therapy" OR "Heat ablation" OR "Heat-based ablation" OR Thermotherapy OR "Radiofrequency ablation" OR RF OR "Radiofrequency catheter ablation" OR Radiofrequency OR "Catheter-based radiofrequency therapy" OR "Cryotherapy ablation" OR Cryotherapy OR Cryo-based OR Cryoablation OR Cryoenergy OR Cryoballoon OR Cryocatheter) | 1,394 Articles |
| **Cochrane** | ("Pulsed field ablation" or "Pulsed-field ablation" or PFA or PulseSelect or Medtronic) and ("Atrial Fibrillation" or "Atrial Fibrillations" or Fibrillation or Atrial or "Auricular Fibrillation" or Auricular or "Persistent Atrial Fibrillation" or "Atrial Fibrillation" or "Persistent Atrial" or "Familial Atrial Fibrillation" or "Paroxysmal Atrial Fibrillation" or "Atrial Fibrillation" or "Paroxysmal Atrial" or "Supraventricular Tachycardia" or Supraventricular) and ("thermal ablation" or thermal or "Heat therapy" or "Heat ablation" or "Heat-based ablation" or Thermotherapy or "Radiofrequency ablation" or RF or "Radiofrequency catheter ablation" or Radiofrequency or "Catheter-based radiofrequency therapy" or "Cryotherapy ablation" or Cryotherapy or Cryo-based or Cryoablation or Cryoenergy or Cryoballoon or Cryocatheter) | 119 Articles |

**Supplementary table 2.** Baseline characteristics of the included studies.

| **Study, year** | **study group** | **Patients, n** | **Age (Years)** | **BMI** | **Sex (Male) N (% )** | **Paroxysmal AF N (% )** | **Persistent AF N (% )** | **LVEF N (% )** | **Total AAD N (% )** | **AAD Class I N (% )** | **AAD Class II N (% )** | **AAD Class III N (% )** | **Hypertension N (% )** | **Diabetes N (% )** | **Coronary artery disease N (% )** | **cerebrovascular disease N (% )** | **heart failure N (% )** | **COPD/Asthma N (% )** | **Obstructive sleep apnea N (% )** | **CHA2DS2-VASc Score, median [IQR]** |
| --- | --- | --- | --- | --- | --- | --- | --- | --- | --- | --- | --- | --- | --- | --- | --- | --- | --- | --- | --- | --- |
| **Badertscher 2023^13^** | PFA | 106 | 64.9 (9.42) | 27.33 (3.76) | 67 (63 ) | 65 (61 ) | 41 (39 ) | 57 (8.6) | NA | NA | NA | NA | 64 (60.4 ) | 12 (11.3 ) | 9 (8.5 ) | NA | NA | NA | NA | NA |
|  | CBA | 75 | 63.5 (9.97) | 26.67 (3.78) | 48 (64 ) | 51 (68 ) | 24 (32 ) | 58 (9.1) | NA | NA | NA | NA | 35 (46.7 ) | 6 (8.0 ) | 6 (8.0 ) | NA | NA | NA | NA | NA |
| **Cochet 2021^16^** | PFA | 18 | 58 (9) | NA | 15 (83 ) | NA | NA | 62 (6) | 14 (77) | 9 (50) | 8 (44) | 5 (28) | 4 (22 ) | 1 (6 ) | 1 (6 ) | 2 (11 ) | NA | NA | NA | NA |
|  | Thermal ablation | 23 | 59 (9) | NA | 17 (74 ) | NA | NA | 61 (8) | 17 (74) | 7 (30) | 9 (39) | 10 (43) | 9 (17 ) | 0 | 2 (9 ) | 1 (4 ) | NA | NA | NA | NA |
| **Maurhofer 2023^10^** | PFA | 40 | 62.50 (9.46) | 25.87 (4.08) | 30 (75 ) | NA | NA | 58.33 (3.84) | NA | NA | NA | NA | 26 (65 ) | 3 (7.5 ) | 8 (20 ) | 2 (5 ) | NA | NA | NA | NA |
|  | CBA | 80 | 62.53 (12.08) | 26.27 (3.47) | 58 (72.5 ) | NA | NA | 60.27 (6.95) | NA | NA | NA | NA | 50 (62.5 ) | 8 (10.0 ) | 14 (17.5 ) | 4 (5 ) | NA | NA | NA | NA |
|  | RFA | 80 | 62.37 (10.80) | 25.90 (3.70) | 63 (78.8 ) | NA | NA | 58.33 (3.77) | NA | NA | NA | NA | 48 (60 ) | 9 (11.2 ) | 10 (12.5 ) | 8 (10 ) | NA | NA | NA | NA |
| **Rattka 2024^37^** | PFA | 94 | 63 (12) | NA | 58 (62 ) | 53 (56 ) | 28 (30 ) | NA | 28 (30) | 22 (24) | NA | 6 (6) | 73 (78 ) | 16 (17 ) | NA | 7 (7 ) | NA | NA | NA | 2.75 (0.6) |
|  | CBA | 47 | 64 (12) | NA | 35(74 ) | 24 (51 ) | 18 (38 ) | NA | 16 (34) | 11 (23) | NA | 5 (11) | 26 (55 ) | 15 (32 ) | NA | 2 (4 ) | NA | NA | NA | 2.75 (0.68) |
| **Della Rocca 2023^11^** | PFA | 174 | 62 (11.6) | 27 (4.8) | 110 (63.2 ) | NA | NA | 59.4 (4.2) | NA | NA | NA | NA | 78 (44.8 ) | 16 (9.2 ) | 10 (5.7 ) | 8 (4.6 ) | 13 (7.5 ) | NA | NA | 2 (0.37) |
|  | CBA | 348 | 62.1 (12.3) | 27.2 (4.6) | 218 (62.6 ) | NA | NA | 58.2 (7.5) | NA | NA | NA | NA | 150 (43.1 ) | 24 (6.9 ) | 24 (6.9 ) | 17 (4.9 ) | 31 (8.9 ) | NA | NA | 2 (0.34) |
|  | RFA | 348 | 62.9 (10.1) | 25.8 (6.7) | 217 (62.4 ) | NA | NA | 57.9 (6.9) | NA | NA | NA | NA | 146 (42 ) | 34 (9.8 ) | 22 (6.3 ) | 14 (4.0 ) | 29 (8.3 ) | NA | NA | 2 (0.34) |
| **Schipper 2023^20^** | PFA | 54 | 69 (11) | 27.8 (5.0) | 37 (69 ) | 16 (30 ) | NA | 53.3 (10.9) | 18 (33) | 1 (2) | NA | 17 (31) | 39 (72 ) | 9 (17 ) | 17 (31 ) | NA | NA | NA | NA | 3.0 (1.8) |
|  | CBA | 54 | 67 (13) | 28.1 (4.5) | 37 (69 ) | 17 (31 ) | NA | 54.9 (10.6) | 15 (28) | 2 (4) | NA | 13 (24) | 37 (69 ) | 9 (17 ) | 14 (26 ) | NA | NA | NA | NA | 2.7 (1.7) |
| **Urbanek 2023^21^** | PFA | 200 | 70 (11.2) | 27.33 (5.23) | 118 (59 ) | 116 (58 ) | NA | NA | NA | NA | NA | NA | 132 (66 ) | 28 (14 ) | 28 (14 ) | 10 (5 ) | 27 (13.5 ) | NA | NA | 2.5 (0.37) |
|  | CBA | 200 | 67.67 (14.19) | 27.67 (4.48) | 108 (54 ) | 127 (63.5 ) | NA | NA | NA | NA | NA | NA | 141 (70.5 ) | 33 (16.5 ) | 27 (13.5 ) | 15 (7.5 ) | 23 (11.5 ) | NA | NA | 2.75 (0.55) |
| **Van de kar 2023^22^** | PFA | 473 | 64.67 (9.67) | 26.9 (3.79) | 301 (63.6 ) | 289 (61.1 ) | 182 (38.5 ) | 55 (11.6) | NA | NA | NA | NA | NA | 44 (9.3 ) | NA | NA | NA | NA | NA | NA |
|  | CBA | 1241 | 63.67 (9.65) | 27.03 (3.79) | 859 (69.2 ) | 818 (65.9 ) | 422 (34.0 ) | 55 (4) | NA | NA | NA | NA | NA | 90 (7.3 ) | NA | NA | NA | NA | NA | NA |
| **Wahedi 2024^23^** | PFA | 50 | 67 (10.6) | 26.2 (3.5) | 32 (64 ) | 36 (72 ) | NA | NA | 15 (30) | NA | NA | NA | 30 (60 ) | 3 (6 ) | NA | 2 (4 ) | 5 (10 ) | 1 (2 ) | 4 (8 ) | 2.2 (1.5) |
|  | CBA | 50 | 65 (10.6) | 29.4 (8.6) | 29 (58 ) | 29 (58 ) | NA | NA | 12 (24) | NA | NA | NA | 35 (70 ) | 8 (16 ) | NA | 3 (6 ) | 5 (10 ) | 2 (4 ) | 5 (10 ) | 2.4 (1.3) |
| **Chaumont 2024^15^** | PFA | 151 | 61 (12) | 27.1 (4.6) | 94 (62 ) | 111 (74 ) | 40 (26 ) | NA | 92 (61) | 53 (35) | NA | 39 (25.8) | 58 (38 ) | 13 (9 ) | 9 (6 ) | 5 (3 ) | 8 (5 ) | NA | 23 (15 ) | 1.13 (0.28) |
|  | CBA | 150 | 63 (12) | 26.9 (4.2) | 93 (62 ) | 109 (73 ) | 41 (27 ) | NA | 90 (60) | 34 (23) | NA | 56 (37.3) | 61 (41 ) | 11 (7 ) | 6 (4 ) | 10 (7 ) | 3 (2 ) | NA | 17 (11 ) | 1.75 (0.19) |
| **Kueffer 2024^17^** | PFA | 214 |  | 28.43 (5.52) | 164 (76.6 ) | NA | NA | 53.33 (11.20) | 76 (40.0) | NA | NA | NA | 130 (60.7 ) | 39 (18.2 ) | 44 (20.6 ) | 12 (5.6 ) | NA | 11 (5.1 ) | 41 (19.2 ) | 2.5 (0.36) |
|  | CBA | 190 | 66.73 (11.95) | 28.93 (5.68) | 138 (72.6 ) | NA | NA | 53.33 (11.21) | 57 (26.6) | NA | NA | NA | 129 (67.9 ) | 21 (11.1 ) | 32 (16.8 ) | 20 (10.5 ) | NA | 4 (2.1 ) | 25 (13.2 ) | 2.5 (0.37) |
|  | RFA | 129 | 66.57 (10.35) | 28.73 (5.25) | 90 (69.8 ) | NA | NA | 53.17 (11.62) | 47 (36.4) | NA | NA | NA | 77 (59.7 ) | 25 (19.4 ) | 24 (18.6 ) | 9 (7 ) | NA | 5 (3.9 ) | 12 (9.3 ) | 2.75 (0.58) |
| **Jungen 2024^24^** | PFA | 74 | 54.33 (32.51) | NA | 46 (62 ) | 38 (51 ) | 36 (49 ) | 49.33 (17.39) | 23 (31) | 18 (24) | NA | 5 (7 ) | 59 (80 ) | 16 (22 ) | 25 (34 ) | 5 (7 ) | NA | NA | NA | 2.6 (1.5) |
|  | CBA | 41 | 65.67 (12.29) | NA | 30 (73 ) | 21 (51 ) | 20 (49 ) | NA | 15 (36.6) | 10 (24) | NA | 5 (12 ) | 25 (61 ) | 13 (32 ) | 15 (37 ) | 2 (5 ) | NA | NA | NA | 2.8 (1.8) |
| **Calvert 2024^25^** | PFA | 208 | 63 (12.69) | 29.33 (5.23) | 136 (65.4 ) | 116 (55.8 ) | NA | NA | 70 (33.7) | NA | NA | NA | 86 (41.3 ) | 14 (6.7 ) | NA | 15 (7.2 ) | 35 (16.8 ) | NA | NA | 2 (0.36) |
|  | CBA | 325 | 63.67 (9.68) | 29 (4.47) | 209 (64.3 ) | 190 (58.5 ) | NA | NA | 139 (42.8) | NA | NA | NA | 150 (46.2 ) | 27 (8.3 ) | NA | 17 (5.2 ) | 42 (12.9 ) | NA | NA | 2.5 (0.35) |
|  | RFA | 174 | 63.67 (10.47) | 29 (4.49) | 116 (66.7 ) | 108 (62.1 ) | NA | NA | 52 (29.9) | NA | NA | NA | 87 (50 ) | 13 (7.5 ) | NA | 8 (4.6 ) | 29 (16.7 ) | NA | NA | 3 (0.37) |
| **Katov 2024^26^** | PFA | 50 | 69.67 (11.45) | 28.53 (2.14) | 28 (56 ) | NA | NA | 57.33 (12.98) | NA | NA | NA | NA | 40 (80 ) | 11 (22 ) | 24 (48 ) | 4 (8 ) | NA | NA | 2 (4 ) | 3.75 (0.67) |
|  | CBA | 50 | 72 (10.69) | 27.13 (5.27) | 26 (52 ) | NA | NA | 56.67 (14.50) | NA | NA | NA | NA | 37 (74 ) | 5 (10 ) | 25 (50 ) | 6 (12 ) | NA | NA | 4 (8 ) | 3.5 (0.45) |
|  | RFA | 43 | 61.9 (9.4) | NA | 31 (72.1 ) | NA | NA | 60.4 (5.8) | NA | NA | NA | NA | 20 (46.5 ) | 2 (4.7 ) | 6 (14 ) | 3 (7.0 ) | NA | NA | NA | NA |
| **My 2023^18^** | PFA | 28 | 69 (12) | NA | 17 (60 ) | NA | NA | 47.4 (12.9) | NA | NA | NA | NA | NA | NA | NA | NA | NA | NA | NA | NA |
|  | RFA | 32 | 65 (13) | NA | 17 (53 ) | NA | NA | 57.6 (4.8) | NA | NA | NA | NA | NA | NA | NA | NA | NA | NA | NA | NA |
| **Osmancik 2023^36^** | PFA | 33 | 60.5 (12.7) | 28.9 (4.9) | 21(63.6 ) | 20 (60.6 ) | 10 (30.3 ) | 58.7 (4.8) | 15 (45.5) | NA | NA | NA | 22 (66.7 ) | 6 (18.2 ) | 4 (12.1 ) | 3 (9.1 ) | NA | NA | NA | 2.39 (1.68) |
|  | RFA | 32 | 64 (10.7) | 30.5 (5.2) | 25(78.1 ) | 20 (62.5 ) | 9 (28.1 ) | 58.2 (4.9) | 17 (53.1) | NA | NA | NA | 22 (68.8 ) | 10 (31.3 ) | 2 (6.3 ) | 1 (3.1 ) | NA | NA | NA | 2.28 (1.69) |
| **Popa 2023^19^** | PFA | 35 | 61.6 (11.4) | 26.7 (4.9) | 24 (68.6 ) | NA | NA | 58.5 (2.7) | NA | NA | NA | NA | 14 (40.0 ) | 1 (2.9 ) | NA | 4 (11.4 ) | NA | NA | NA | 1.7 (1.7) |
|  | RFA | 144 | 63.43 (10.06) | 26.56 (4.2) | 86 (59.7) | NA | NA | 58.51 (5.38) | NA | NA | NA | NA | 78 (54.2) | 11 (7.64) | NA | 7 (4.86) | NA | NA | NA | 1.96 (1.56) |
| **De Becker 2024^14^** | PFA | 161 | 65 (8.98) | 27 (3.74) | 103 (64 ) | 125 (78 ) | 36 (22 ) | 151 (94 ) | 103 (64) | NA | NA | NA | 80 (50 ) | 14 (9 ) | NA | NA | NA | NA | NA | 2 (0.38) |
|  | RFA | 161 | 65.33 (9.72) | 27 (2.99) | 101 (63 ) | 123 (76 ) | 38 (24 ) | 146 (91 ) | 87 (54) | NA | NA | NA | 77 (48 ) | 15 (9 ) | NA | NA | NA | NA | NA | 2 (0.38) |
| **Osmancik 2024^34^** | PFA | 47 | 63.3 (9.9) | 30 (5.1) | 28 (59.6 ) | 23 (48.9 ) | 21 (44.7 ) | 59 (5.7) | 26 (55.3) | 17 (36.2) | NA | 8 (17.0) | 37 (78.7 ) | 9 (19.1 ) | 4 (8.5 ) | 4 (8.5 ) | NA | NA | NA | 2.38 (1.57) |
|  | RFA | 23 | 67.7 (10.4) | 28.3 (4.2) | 9 (39.1 ) | 13 (56.5 ) | 10 (43.5 ) | 58.3 (9.7) | 10 (43.5) | 5 (21.7) | NA | 5 (21.7) | 17 (73.9 ) | 3 (13 ) | 1 (4.3 ) | 0 | NA | NA | NA | 2.78 (1.51) |
| **Reinsch 2024^35^** | PFA | 201 | 66.67 (10.45) | 27.33 (3.73) | 114 (56 ) | NA | NA | NA | 46 (23) | NA | NA | NA | 133 (66 ) | 22 (11 ) | 19 (9 ) | 11 (5 ) | NA | NA | NA | 2.25 (0.55) |
|  | HPSD-RF | 209 | 67.33 (11.94) | 27.67 (4.48) | 111 (53 ) | NA | NA | NA | 43 (21) | NA | NA | NA | 141 (67 ) | 27 (13 ) | 21 (10 ) | 22 (11 ) | NA | NA | NA | 2 (0.36) |
| **Popa 2024^27^** | PFA | 145 | 65.7 (9.8) | 28.1 (5.5) | 104 (71.7 ) | NA | 75 (51.7 ) | NA | NA | NA | NA | NA | 77 (55.8 ) | 17 (12.4 ) | NA | 13 (9.5 ) | 19 (13.9 ) | NA | NA | 2 (0.38) |
|  | RFA | 70 | 65.6 (8.6) | 28.1 (4.8) | 45 (64.3 ) | NA | 33 (47.1 ) | NA | NA | NA | NA | NA | 39 (55.7 ) | 12 (17.1 ) | NA | 3 (4.3 ) | 12 (17.1 ) | NA | NA | 2 (0.42) |
| **Soubh 2024^29^** | PFA | 52 | 67 (10) | 28 (6) | 37 (71 ) | 25 (48 ) | 27 (52 ) | 52 (8) | 14 (27) | NA | NA | NA | 43 (83 ) | 8 (15 ) | 9 (17 ) | 5 (10 ) | 20 (38 ) | 5 (10 ) | 8 (15 ) | 4 (1.6) |
|  | vHPSD-RF | 30 | 73 (7) | 27 (6) | 14 (47 ) | 13 (43 ) | 17 (57 ) | 47 (14) | 5 (17) | NA | NA | NA | 25 (83 ) | 4 (13 ) | 13 (43 ) | 4 (13 ) | 13 (43 ) | 4 (13 ) | 5 (17 ) | 4 (1.7) |
| **Wormann 2023^28^** | PFA | 57 | 67 (13) | 28 (5) | 19 (33 ) | 17 (30 ) | 40 (70 ) | 56 (6) | 16 (28) | NA | NA | NA | 37 (65 ) | 9 (16 ) | 14 (25 ) | NA | NA | NA | 6 (11 ) | NA |
|  | VHPSD-RF | 57 | 67 (12) | 27 (4) | 23 (40 ) | 17 (30 ) | 40 (70 ) | 56 (9) | 13 (23) | NA | NA | NA | 34 (60 ) | 8 (14 ) | 11 (19 ) | NA | NA | NA | 7 (12 ) | NA |
| **Pannone 2024^30^** | PFA | 80 | 68.1 (8.6) | NA | 58 (72.5) | NA | NA | 54 (7.9) | 67 (83.8) | 19 (23.8) | 42 (52.5) | 22 (27.5) | 59 (73.8) | 17 (21.2) | 11 (13.8) | 1 (1.2) | 10 (12.5) | NA | NA | 2.1 (1.1) |
|  | CBA | 80 | 67.1 (9) | NA | 54 (67.5) | NA | NA | 55.1 (8.2) | 65 (81.2) | 16 (20) | 47 (58.8) | 23 (28.8) | 56 (70) | 12 (15) | 11 (13.8) | 1 (1.2) | 8 (10) | NA | NA | 1.9 (1.3) |
| **Nakasone 2025^31^** | PFA | 221 | 80 (4) | 27.3 (4.6) | 127 (57.5) | 88 (39.8) | 109 (49.3) | 57.3 (7.5) | NA | NA | NA | NA | 153 (69.2) | 25 (11.3) | 35 (15.8) | 17 (7.7) | NA | NA | NA | 3.6 (1.1) |
|  | CBA | 216 | 80 (3) | 26.9 (3.6) | 107 (49.5) | 120 (55.6) | 96 (44.4) | 58.3 (7.8) | NA | NA | NA | NA | 156 (72.2) | 24 (11.1) | 46 (21.3) | 14 (6.5) | NA | NA | NA | 3.8 (1.1) |
|  | RFA | 546 | 80 (3) | 27.8 (5.3) | 330 (60.4) | 189 (34.6) | 309 (56.6) | 57.3 (8.7) | NA | NA | NA | NA | 329 (60.3) | 80 (14.7) | 123 (22.5) | 18 (3.3) | NA | NA | NA | 3.5 (1.1) |
| **Wang 2024^32^** | PFA | 27 | 65.48 (10.72) | 24.03 (3.53) | 16 (59) | NA | NA | 59.07 (2.97) | 19 (70.37) | 1 (4) | 14 (52) | 1 (4) | 12 (44) | 6 (22) | 6 (22) | 3 (11) | 0 | NA | NA | NA |
|  | CBA | 41 | 61.68 (11.04) | 25.43 (3.56) | 25 (61) | NA | NA | 59.2 (1.89) | 28 (68.29) | 1 (2) | 16 (39) | 9 (22) | 20 (49) | 2 (5) | 10 (24) | 4 (10) | 2 (5) | NA | NA | NA |
|  | RFA | 41 | 57.78 (11.31) | 23.69 (2.73) | 27 (66) | NA | NA | 58.18 (2.42) | 19 (46.34) | 0 | 11 (27) | 3 (7) | 56 (56) | 4 (10) | 4 (10) | 1 (2) | 0 | NA | NA | NA |
| **Zhang 2025^33^** | PFA | 19 | 62 (5.61) | 25.2 (4.3) | 9 (47.4) | NA | NA | 62.3 (2.2) | NA | NA | NA | NA | 13 (68.4) | 4 (21.1) | 4 (21.1) | 5 (26.3) | NA | 0 | NA | 2.75 (0.81) |
|  | CBA | 127 | 63.67 (11.25) | 24 (3.2) | 69 (54.3) | NA | NA | 61 (4.4) | NA | NA | NA | NA | 80 (63) | 20 (15.7) | 37 (29.1) | 22 (17.3) | NA | 4 (3.1) | NA | 2.25 (0.58) |
|  | RFA | 77 | 72.67 (29.46) | 24.2 (2.2) | 41 (53.2) | NA | NA | 61.4 (4.9) | NA | NA | NA | NA | 44 (57.1) | 9 (11.7) | 24 (31.2) | 10 (13) | NA | 4 (5.2) | NA | 2 (0.42) |

**Supplementary Table. 3 Periprocedural complications reported in the included studies**

| **Study ID** | **Transient PNP** | **Persistent PNP** | **Cardiac tamponade** | **Esophageal injury** | **Groin complications** | **Vascular access complications** | **Thromboembolic events** |
| --- | --- | --- | --- | --- | --- | --- | --- |
| **Baderscher 2023** | NR | NR | 2/0 | NR | NR | 0/0 | 0/0 |
| **Rattka 2024** | 0/1 | NR | 1/0 | NR | NR | NR | 2/0 |
| **Schipper 2023** | 0/2 | NR | 2/0 | 0/0 | 0/2 | NR | 0/1 |
| **Urbanek 2023** | 2/12 | 0/3 | 1/0 | 0/1 | NR | 5/7 | 0/1 |
| **Van de kar 2023** | 0/9 | 0/6 | 3/4 | NR | 1/15 | NR | 1/3 |
| **Wahedi 2024** | 0/1 | NR | 1/0 | 0/0 | 1/0 | NR | 0/0 |
| **Chaumont 2024** | NR/13 | 0/2 | 1/0 | NR | NR | 0/1 | 0/0 |
| **Jungen 2024** | NR | NR | 2/0 | NR | 1/0 | NR | NR |
| **Katov 2024** | 0/2 | 0/0 | 0/0 | NR | NR | 0/0 | 0/0 |
| **My 2023** | NR | NR | NR | NR | NR | NR | NR |
| **Osmancik 2023** | 0/0 | 0/0 | 0/0 | NR | NR | 0/0 | 0/0 |
| **Popa 2023** | NR | NR | 0/0 | 0/0 | 5/9 | NR | 2/1 |
| **De Becker 2024** | NR | NR | 0/0 | 0/1 | NR | 0/0 | 1/0 |
| **Osmancik 2024** | NR | NR | NR | NR | NR | NR | NR |
| **Reinsch 2024** | NR | NR | 3/6 | NR | NR | 1/3 | 2/2 |
| **Popa 2024** | NR | NR | NR | NR | NR | NR | NR |
| **Soubh 2024** | NR | NR | 0/0 | 0/0 | NR | 0/0 | 0/1 |
| **Wormann 2023** | NR | NR | 2/0 | NR | 0/2 | NR | NR |
| **Cochet 2021** | 0/0 | 0/0 | NR | 0/10 | 1/2 | NR | NR |
| **Maurhofer 2023** | 0/0 | 0/0 | 2/0 | 0/0 | NR | NR | 0/0 |
| **Della Rocca 2023** | 0/17 | 0/1 | 0/2 | 0/0 | 4/11 | 1/3 | 1/1 |
| **Kueffer 2024** | 0/0 | 0/5 | 2/0 | 0/0 | NR | 3/1 | 0/0 |
| **Calvert 2024** | 0/5 | 0/2 | 0/0 | NR | NR | 0/2 | 0/0 |
| **Zhang 2025** | NR | NR | 0/0 | NR | NR | NR | 0/5 |
| **Nakasone 2025** | 0/11 | 0/5 | 0/3 | NR | 1/10. | NR | 0/1 |
| **Wang 2024** | 0/1 | 0/0 | 0/0 | 0/0 | NR | NR | NR |
| **Pannone 2024** | 0/7 | NR | NR | NR | NR | 0/0 | 0/0 |

**Supplementary table. 4** NOS for the included case‒control studies.

| **Study ID** | **Selection (maximum 4)** | | | | **Outcomes selected** | **Comparability (maximum 2)** | **Outcome (maximum 3)** | | | **Total score (maximum 9)** | **Overall appraisal (poor, fair, or good)** |
| --- | --- | --- | --- | --- | --- | --- | --- | --- | --- | --- | --- |
|  |  |  |  |  |  |  |  |  |  |  |  |
|  | **1** | **2** | **3** | **4** | **5** | | **6** | **7** | **8** |  |  |
|  | **Is the case definition adequate?** | **Representativeness of the cases** | **Selection of Controls** | **Definition of controls** | **Comparability of cohorts on the basis of the design or analysis** | | **Ascertainment of exposure** | **Same method of ascertainment for cases and controls** | **Non-Response rate** |  |  |
|  |  |  |  |  |  |  |  |  |  |  |  |
|  | **(0 or ✵)** | **(0 or ✵)** | **(0 or ✵)** | **(0 or ✵)** | **(0, ✵, or ✵✵)** | | **(0 or ✵)** | **(0 or ✵)** | **(0 or ✵)** |  |  |
| **Rattka 2024** | ✵ | ✵ | ✵ | ✵ | Total procedure duration, Fluoroscopy time | ✵ | ✵ | ✵ | ✵ | 8 | GOOD |

Supplementary Table. 5 Assessment of Potentially Overlapping Cohorts.

| Study pair | Registry/Institution | Recruitment Periods | Potential Overlap | Decision and Rationale |
| --- | --- | --- | --- | --- |
| Wörmann et al., 2023 and Schipper et al., 2023 | University of Cologne, Germany | Both included patients enrolled between September 2021 and October 2022 | High | These studies likely included overlapping PFA patients from the same institution and recruitment period. Therefore, only one study was included for outcomes reported by both studies to avoid double counting |
| Rattka et al., 2024 and Jungen et al., 2024 | University Hospital Essen, Germany | Not fully reported | Possible | Both studies originated from the same institution; however, they investigated different patient populations and reported different outcomes. Overlap was considered unlikely to materially affect pooled estimates. |
| Maurhofer et al., 2024 and Badertscher et al., 2023 | SWISS-AF-PVI Registry, Bern University Hospital, Switzerland | Partially overlapping periods | Possible | Maurhofer et al. included only paroxysmal AF patients and used propensity-score matching, whereas Badertscher et al. included both paroxysmal and persistent AF patients. Both studies were retained, but overlapping populations were carefully considered during outcome selection. |
| Kueffer et al., 2024 and Maurhofer et al., 2024 | SWISS-AF-PVI Registry, Bern University Hospital, Switzerland | Partially overlapping periods | Low | Kueffer et al. exclusively enrolled patients with persistent AF, whereas Maurhofer et al. included only paroxysmal AF patients. Therefore, patient overlap was considered unlikely. |
| Kueffer et al., 2024 and Badertscher et al., 2023 | SWISS-AF-PVI Registry, Bern University Hospital, Switzerland | Partially overlapping periods | Possible | Differences in study populations and outcome reporting reduced the likelihood of substantial overlap affecting pooled analyses. Studies were retained with careful outcome-level assessment. |

Supplementary Table. 6 Blanking period of the included studies

| Study | Blanking Period |
| --- | --- |
| **Badertscher 2023** | 3 Months |
| Cochet 2021 | NA |
| **Maurhofer 2023** | 3 Months |
| **Rattka 2024** | 3 Months |
| **Della Rocca 2023** | 3 Months |
| **Schipper 2023** | 3 Months |
| **Urbanek 2023** | 3 Months |
| **Van de kar 2023** | NA |
| **Wahedi 2024** | NA |
| **Chaumont 2024** | 3 Months |
| **Kueffer 2024** | 3 Months |
| **Jungen 2024** | 3 Months |
| **Calvert 2024** | 2 Months |
| **Katov 2024** | NA |
| **My 2023** | NA |
| **Osmancik 2023** | NA |
| **Popa 2023** | 2 Months |
| **De Becker 2024** | 3 Months |
| **Osmancik 2024** | NA |
| **Reinsch 2024** | NA |
| **Popa 2024** | NA |
| **Soubh 2024** | 3 Months |
| **Wormann 2023** | 3 Months |
| **Pannone 2024** | 3 Months |
| **Nakasone 2025** | 3 Months |
| **Wang 2024** | 3 Months |
| **Zhang 2025** | NA |

**Supplementary figure. 1** ROBINS-I for the included nonrandomized studies.

**Supplementary figure. 2** ROB-2 for the included randomized study.

**Supplementary figure. 3** Kaplan-Meier curve estimates of freedom from tachyarrhythmia recurrence in the PFA group vs thermal ablation group in persistent AF patients.

**Supplementary figure. 4** Kaplan-Meier curve estimates of freedom from tachyarrhythmia recurrence for PFA vs CBA vs RFA.

**Supplementary Figure. 5** Forest plot of AF recurrence for PFA vs thermal ablation.

**Supplementary Figure. 6** Labbe plot of AF recurrence for PFA vs thermal ablation.

**Supplementary Figure. 7** Forest plot of durable PVI for PFA vs thermal ablation.

**Supplementary Figure. 8** Forest plot of durable PVI for PFA vs thermal ablation.

**Supplementary Figure. 9** Forest plot of redo procedure for PFA vs thermal ablation.

**Supplementary Figure. 10** Leave-one-out sensitivity analysis of redo procedure for PFA vs thermal ablation.

**Supplementary Figure. 11** Forest plot of redo procedure for PFA vs thermal ablation.

**Supplementary Figure. 12** Forest plot of transient PNP for PFA vs thermal ablation.

**Supplementary Figure. 13** Forest plot of transient PNP for PFA vs thermal ablation.

**Supplementary Figure. 14** Forest plot of persistent PNP for PFA vs thermal ablation.

**Supplementary Figure. 15** Forest plot of persistent PNP for PFA vs thermal ablation.

**Supplementary Figure. 16** Forest plot of cardiac tamponade for PFA vs thermal ablation.

**Supplementary Figure. 17** Forest plot of cardiac tamponade for PFA vs thermal ablation.

**Supplementary Figure. 18** Forest plot of esophageal injury for PFA vs thermal ablation.

**Supplementary Figure. 19** Forest plot of esophageal injury for PFA vs thermal ablation.

**Supplementary Figure. 20** Forest plot of groin complication for PFA vs thermal ablation.

**Supplementary Figure. 21** Forest plot of groin complications for PFA vs thermal ablation.

**Supplementary Figure. 22** Forest plot of VAC for PFA vs thermal ablation.

**Supplementary Figure. 23** Forest plot of VAC for PFA vs thermal ablation.

**Supplementary Figure. 24** Forest plot of thromboembolic complications for PFA vs thermal ablation.

**Supplementary Figure. 25** Forest plot of thromboembolic complications for PFA vs thermal ablation.

**Supplementary Figure. 26** Forest plot of procedure duration for PFA vs thermal ablation.

**Supplementary Figure. 27** Leave-one-out sensitivity analysis of procedure duration for PFA vs thermal ablation.

**Supplementary Figure. 28** Forest plot of procedure duration for PFA vs thermal ablation.

**Supplementary Figure. 29** Forest plot of skin-to-skin duration for PFA vs thermal ablation.

**Supplementary Figure. 30** leave-one-out sensitivity analysis of skin-to-skin duration for PFA vs thermal ablation.

**Supplementary Figure. 31** Forest plot of skin-to-skin duration for PFA vs thermal ablation.

**Supplementary Figure. 32** Forest plot of LA dwell time for PFA vs thermal ablation.

**Supplementary Figure. 33** Leave-one-out sensitivity analysis of LA dwell time for PFA vs thermal ablation.

**Supplementary Figure. 34** Forest plot of LA dwell time for PFA vs thermal ablation.

**Supplementary Figure. 35** Forest plot of Fluoroscopy time for PFA vs thermal ablation.

**Supplementary Figure. 36** Leave-one-out sensitivity analysis of fluoroscopy time for PFA vs thermal ablation.

**Supplementary Figure. 37** Forest plot of Fluoroscopy time for PFA vs thermal ablation.
